# Advances in improving cancer immunotherapy with nanotechnology: from smart nanoparticles to synergistic combination strategies

**DOI:** 10.1186/s12943-026-02736-0

**Published:** 2026-07-28

**Authors:** Untack Cho, Jingjing Pu, Amit Sharma, Junhyuk Song, Ji Hyeon You, Yona Kim, Pyung Won Im, Hyung Woo Park, Hyo Eun Moon, Ho Sung Myeong, Hye Ran Park, Jian Hou, Ingo G. H. Schmidt-Wolf, Sun Ha Paek

**Affiliations:** 1 Department of Neurosurgery, Cancer Research Institute and Ischemic/Hypoxic Disease Institute, Seoul National University College of Medicine, Seoul, Korea; 2 Department of Life and Chemical Engineering, Gyeonggi University of Science and Technology, Siheung, Korea; 3 Department of Hematology, Renji Hospital, Shanghai Jiao Tong University School of Medicine, Shanghai, China; 4 Department of Integrated Oncology, Center for Integrated Oncology (CIO) Bonn, University Hospital Bonn, Bonn, Germany; 5 Department of Neurosurgery, Soonchunhyang University Seoul Hospital, Seoul, 04401 Republic of Korea; 6 Advanced Institute of Convergence Technology, Seoul National University, Suwon, Korea

**Keywords:** Cancer Immunotherapy, Nanotechnology, Tumor Microenvironment, Drug Delivery, Nanovaccines, Cytokine-induced killer (CIK) cell immunotherapy

## Abstract

Cancer immunotherapy has substantially advanced cancer treatment, achieving durable responses in select malignancies. However, its widespread application is limited by significant challenges: low efficacy in many solid tumors, severe side effects, and immune evasion facilitated by the tumor microenvironment (TME). Nanotechnology offers a promising approach to address these obstacles. By employing nanoparticles (NPs), we can precisely deliver therapeutics to tumor sites, ensure controlled release to minimize side effects, and amplify the immune response, thereby substantially boosting the effectiveness of immunotherapy. This review comprehensively highlights the latest advancements in using nanotechnology to enhance cancer immunotherapy. This paper details various applications of nanotech in this field. It discusses smart nanoparticles that respond to TME signals to release drugs (e.g., checkpoint inhibitors) directly at the tumor, reducing systemic side effects and activating T-cells. We also explore how nanovaccines, which co-deliver tumor markers and immune boosters, can induce antigen-specific immune responses. Furthermore, mRNA-loaded nanoparticles can directly modify CAR T-cells inside the body, simplifying treatment and increasing efficacy. Strategies like using PLGA NPs to deliver immune enhancers such as IL-2 are also presented, which activate immune cells while minimizing systemic issues. The review also explains how nanoparticles can re-engineer the immunosuppressive TME to create an environment more conducive to immune action. We also emphasize that nanotechnology-enhanced adoptive therapies, particularly cytokine-induced killer (CIK) cell immunotherapy, hold great potential to improve tumor targeting, treatment persistence durability, and overall anticancer efficacy. Collectively, we highlight synergistic effects achieved by combining nanoparticles with other treatments like chemotherapy, radiation, photothermal/photodynamic therapy, and more, which can turn hard-to-treat tumors into susceptible targets. The integration of nanotechnology and immunotherapy holds the potential to meaningfully advance future cancer therapy.

## Introduction

### Overview of cancer immunotherapy

Cancer remains one of the leading causes of mortality worldwide, with millions of new cases and deaths reported each year [1]. Traditional cancer treatments such as surgery, chemotherapy, and radiation therapy have contributed to improved patient outcomes in certain settings, but their non-specific nature and dose-limiting toxicities often compromise both efficacy and quality of life [2]. Over the past two decades, cancer immunotherapy has emerged as a meaningful advancement in oncology, offering a new strategy that leverages the body’s own immune system to selectively target and eliminate cancer cells [3]. Cancer immunotherapy has fundamentally reshaped the landscape of oncology, offering a paradigm shift from traditional treatments by harnessing the body's immune system to target malignancies with improved specificity and, in some cases, durable responses compared to conventional therapies [4]. Unlike conventional therapies such as chemotherapy and radiotherapy, which primarily target tumor cells through cytotoxic mechanisms, immunotherapy empowers the immune system to recognize and eliminate cancer cells, often achieving long-term remission and improved patient outcomes [5]. This approach encompasses a range of strategies, each designed to exploit distinct immunological mechanisms to overcome tumor-mediated immune evasion. Immune checkpoint inhibitors, such as antibodies targeting programmed cell death protein 1 (PD-1), its ligand (PD-L1), and cytotoxic T-lymphocyte-associated antigen 4 (CTLA-4), block inhibitory pathways that tumors exploit to suppress T-cell activity, thereby promoting measurable anti-tumor immune responses [6]. Chimeric antigen receptor (CAR) T-cell therapy involves genetically engineering T-cells to express receptors that specifically recognize tumor-associated antigens, enabling precise targeting and destruction of cancer cells [7]. Cancer vaccines stimulate antigen-specific immune responses by delivering tumor antigens and adjuvants to dendritic cells, which prime T-cells for targeted tumor attack [8]. Cytokine therapies, utilizing signaling molecules like interleukin-2 (IL-2) or IL-15, amplify immune cell proliferation and effector functions to enhance anti-tumor immunity [9]. These strategies have led to significant clinical successes, with therapies like ipilimumab (anti-CTLA-4), pembrolizumab (anti-PD-1), and CAR T-cell treatments for hematological malignancies gaining regulatory approval and transforming the standard of care for cancers such as melanoma, lung cancer, and leukemia [10].

Despite these breakthroughs, cancer immunotherapy faces substantial challenges that limit its efficacy and broader applicability. One major hurdle is the low response rate observed in many cancers, particularly solid tumors, where only a subset of patients achieves durable responses due to tumor heterogeneity and complex immune evasion mechanisms [11]. For instance, immune checkpoint inhibitors are effective in tumors with high mutational burdens, such as melanoma and non-small cell lung cancer, but show limited efficacy in immunologically "cold" tumors with low T-cell infiltration [12]. Off-target effects, including immune-related adverse events (irAEs) such as colitis, pneumonitis, and endocrinopathies, pose significant risks, as systemic immune activation can lead to severe toxicity and necessitate treatment discontinuation [13]. The tumor microenvironment (TME) further complicates immunotherapy, as it is often characterized by immunosuppressive cells, including tumor-associated macrophages (TAMs) and myeloid-derived suppressor cells (MDSCs), which dampen immune responses and promote tumor progression [14]. Physical barriers within the TME, such as dense extracellular matrix and aberrant tumor vasculature, hinder the penetration and efficacy of immunotherapeutic agents, reducing their therapeutic impact [15]. Moreover, systemic administration of therapies like cytokines can lead to short half-lives and dose-limiting toxicities, further constraining their clinical utility [16]. These limitations highlight the critical need for innovative strategies to enhance the specificity, potency, and safety of cancer immunotherapy, enabling broader patient access and improved outcomes [17].

### Importance of nanotechnology in enhancing immunotherapy

To address these shortcomings, researchers have increasingly turned to nanotechnology, a field that manipulates materials at the molecular and atomic levels to design nanoparticles (NPs) with unique properties. These include tunable size, surface charge, composition, and functionalization potential, allowing for precise delivery of therapeutic payloads [18]. Nanotechnology has emerged as a versatile platform to address these challenges, offering versatile tools to optimize the delivery, efficacy, and safety of immunotherapeutic agents [19]. By leveraging nanocarriers such as liposomes, polymeric nanoparticles, micelles, exosomes, and inorganic nanoparticles, therapeutic agents can be preferentially delivered to tumor tissues. This targeting is partly facilitated by the enhanced permeability and retention (EPR) effect, which arises from the abnormal vascular architecture and impaired lymphatic drainage characteristic of many solid tumors [20]. This targeted delivery minimizes systemic exposure, reducing off-target toxicity and enhancing the therapeutic index of immunotherapies [21]. For example, nanoparticle-based delivery of immune checkpoint inhibitors, such as anti-PD-1/PD-L1 or anti-CTLA-4 antibodies, has been shown to improve tumor-specific targeting while reducing systemic immune activation, thereby mitigating irAEs [22]. Similarly, nanovaccines enable co-delivery of tumor antigens and adjuvants to dendritic cells, optimizing antigen presentation and eliciting robust, antigen-specific immune responses [23]. In CAR-T cell therapy, nanoparticles offer a less invasive alternative to ex vivo T-cell engineering by delivering genetic material, such as mRNA or CRISPR/Cas9, directly to T-cells in vivo, enhancing the feasibility and scalability of this approach [24]. Cytokine delivery also benefits from nanotechnology, as nanocarriers enable controlled and sustained release of cytokines like IL-2 or IL-15, maintaining therapeutic efficacy while reducing systemic toxicity [25]. Beyond delivery, nanotechnology can modulate the immunosuppressive TME by targeting TAMs and MDSCs or delivering immunomodulatory agents to reprogram the TME into an immune-active state, enhancing the efficacy of immunotherapies [26].

Advances in material science and nanotechnology have led to the development of multifunctional nanocarriers capable of simultaneous drug delivery, imaging, and immune modulation, paving the way for theranostic applications [27]. The integration of nanotechnology with immunotherapy opens new avenues for personalized and precision medicine, enhancing the potential for tailored therapeutic strategies [28]. These advancements underscore the considerable potential of nanotechnology in overcoming the barriers to effective immunotherapy, offering hope for improved clinical outcomes across diverse cancer types [29]. Subsequently, we discuss the clinical translation of nano-immunotherapy, highlighting current clinical trials, regulatory challenges, and safety considerations. We then explore future directions, including emerging nanomaterials, AI-assisted nanoparticle design, integration with gene editing technologies (e.g., CRISPR), and microbiome-targeted approaches. Finally, we present a roadmap for clinical translation, emphasizing the need for interdisciplinary collaboration among immunologists, materials scientists, oncologists, and engineers.

## Advances in nanotechnology-enhanced Immunotherapy

Building on the challenges outlined in the Introduction, this section examines how nanotechnology addresses these limitations across six core immunotherapy modalities. We discuss nanoparticle-based delivery strategies for immune checkpoint inhibitors, cancer vaccines, CAR-T cell therapy, complement-based immunotherapy, cytokine delivery, and cytokine-induced killer (CIK) cell therapy, followed by exploration of tumor microenvironment modulation, magnetic hyperthermia, and antibody–drug conjugate platforms. Conceptual combination strategies that integrate these modalities are also presented to illustrate the synergistic potential of nanotechnology-enhanced cancer immunotherapy.

Given the diversity of nanocarrier systems covered in this section, Tables 1 and Table 2 are introduced here to provide readers with an early point of reference. Table 1 summarizes the major nanoparticle platforms discussed in the review, with emphasis on their representative applications, advantages, limitations, and translational relevance. Table 2 further compares these platforms from a therapeutic perspective, including their payloads, targeting mechanisms, clinical advantages, and key barriers to translation. Together, these tables are intended to make the following sections easier to follow and to clarify how differences in nanocarrier design may influence immune modulation, safety, and clinical development.

**Table 1:** Comparative summary of nanoparticle platforms used in cancer immunotherapy

Nanoparticle Type/Platform	Therapeutic Payloads	Targeting Mechanism	Key Advantages	Key Limitations	Representative Application (Section)
Lipid Nanoparticles (LNPs)	- mRNA (CAR constructs, neoantigens) - Anti-PD-1/PD-L1 antibodies - IL-2, IL-15 cytokines; CRISPR/Cas9	- Passive EPR effect - Active: CD5/CD8/CD19-targeted ligands - pH-responsive ionizable lipids	- > 96% encapsulation; > 80% T-cell transfection - Clinically validated (COVID mRNA vaccines) - Microfluidic GMP manufacturing (CV < 10%)	- Rapid RES clearance (liver/spleen) - Cold-chain storage required - PEG coating immunogenicity	ICI delivery, mRNA vaccines, in vivo CAR-T (Sec. "Immune Checkpoint inhibitor", "Cancer Vaccines", "CAR-T Cell Therapy Enhancement")
Polymeric NPs (PLGA-based)	- Anti-CTLA-4, anti-PD-L1 antibodies - IL-2, IL-12; siRNA (STAT3, MDSC) - STING agonists (ONP-302)	- Passive EPR + PEG coating - pH-responsive degradation (acidic TME) - Folate/antibody fragment conjugation	- FDA-approved polymer; biodegradable - 30% systemic toxicity reduction vs. free Ab - Sustained/controlled multi-drug release	- Batch-to-batch loading variability - Lower nucleic acid transfection vs. LNPs - Residual organic solvent risk	ICI delivery, cytokine therapy, TME modulation (Sec. "Immune Checkpoint inhibitor", "Cytokine Delivery", "Tumor Microenvironment Modulation")
Exosomes/ Extracellular Vesicles (EVs)	- PD-L1 siRNA (> 90% knockdown) - Anti-PD-L1, anti-CD71 antibodies - IFN-gamma mRNA; doxorubicin	- Natural cell-membrane tropism - CD64 Fcgamma receptor surface display - Tumor-derived homotypic targeting	- Biomimetic; low immunogenicity - 35% response rate in Phase I (NCT05191836) - Simultaneous targeting + checkpoint blockade	- Ultracentrifugation purification barriers - Heterogeneous cargo loading - Donor cell variability in batches	Complement immunotherapy, ICI delivery (Sec. "Immune Checkpoint inhibitor", "Nanotechnology in Complement Based Immunotherapy", "CIK Cell Therapy Enhancement")
Inorganic NPs - Gold (AuNPs)	- Anti-PD-L1 antibodies - Cisplatin (chemo-immunotherapy) - Chlorin e6 (photodynamic therapy)	- Active: antibody conjugation - NIR light-triggered photothermal activation - Surface plasmon resonance (theranostics)	- Dual theranostic (CT/PA imaging + therapy) - Synergistic photodynamic + immune activation - Precise surface functionalization	- 6-month liver/spleen retention - Non-biodegradable; unclear clearance - High production cost	Theranostic ICI delivery, photo- immunotherapy (Sec. " Immune Checkpoint inhibitor", " Magnetic Hyperthermia and Immunotherapy: Synergistic Relationships")
Inorganic NPs - Hafnium Oxide (NBTXR3)	- Radioenhancer (crystalline HfO2) - Combined SBRT + anti-PD-1 (pembrolizumab/nivolumab)	- Intratumoral injection + EPR - Radiation-activated radioenhancement - Tumor-selective dose amplification	- ORR 40%, DCR 75% (Phase I, NCT03589339) - HNSCC ORR 31.3%, DOR 14.8 months - Active in prior anti-PD-1-resistant patients	- Requires intratumoral injection - Dependent on radiation co-administration - Long-term inorganic NP retention	Radioenhancer + ICI delivery (Sec. " Nanoparticle Delivered ICIs")
Inorganic NPs - Magnetic (Iron Oxide, Fe3O4)	- Anti-PD-L1/PD-1 antibodies - IL-2, IL-15; CpG oligonucleotides - Doxorubicin/paclitaxel; MRI contrast	- AMF-induced MHT - PEGylation for EPR-enhanced circulation - pH/redox-responsive payload release	- MHT induces ICD—> DC maturation - 50% tumor volume reduction vs. standalone ICI - 2.5-fold CD8 + T-cell infiltration increase	- Long-term iron organ accumulation risk - AMF equipment required (limited infra.) - Heating uniformity challenges	Magnetic hyperthermia + immunotherapy (Sec. "Magnetic Hyperthermia and Immunotherapy: Synergistic Relationship")
Inorganic NPs - Mesoporous Silica/MOF	- Multivalent IgG3 Fc fragments - TLR7 agonist (imiquimod) - Chemotherapeutics; siRNA	- MMP-2-cleavable PEG shield (TME-responsive) - Fc fragment complement activation - Folate/antibody receptor-mediated endocytosis	- MMP-2-triggered complement + macrophage activation - No chronic toxicity at 1 year - High surface area; tunable pore release	- Long-term silica accumulation (lung/liver) - Complex multi-step functionalization - Clearance pathway not fully characterized	Complement activation, TME modulation (Sec. " Cytokine Delivery", " Proposed Synergistic Multimodal Platforms")
Cell Membrane- Coated NPs	- IL-2, IL-15 cytokines - Tumor antigens - Immune checkpoint molecules	- Homotypic targeting via cancer cell membrane - Immune cell membrane coating (immune camouflage) - CD47/PD-L1 'don't eat me' signals	- threefold cytokine delivery increase vs. bare NPs - Inherent immune evasion; reduced RES clearance - Enhanced tumor specificity	- Complex, low-yield membrane extraction - Risk of transferring oncogenic signals - Preclinical stage only; no clinical data	Cytokine delivery, TME modulation (Sec. " Tumor Microenvironment Modulation")
Hybrid/ Multifunctional NPs	- STING agonist + tumor antigens - Fe3O4 core + DOX + anti-HER2/EGFR Ab - TLR4/STING co-agonists (PMM NPs)	- Multi-modal: EPR + active ligand + stimuli-responsive - STING + TLR4 co-activation (NF-kB + IRF3) - AMF + pH/redox dual-responsive release	- 4.0-fold IFN-beta increase (PMM NPs) - Complete rechallenge tumor rejection with anti-PD-1 - Overcomes single-modality resistance	- Highly complex formulation development - Regulatory pathway unclear for multi-component - Preclinical efficacy not yet replicated clinically	TME modulation, MHT + ADC combination (Sec. " CIK Cell Therapy Enhancement", "Tumor Microenvironment Modulation", "ADC anti-cancer action and AMF-mediated immune cell recruitment")
Liposomes (incl. Nano- liposomal)	- Irinotecan (nal-IRI, Onivyde) - Doxorubicin + irinotecan (co-delivery) - Anti-PD-L1 antibodies	- Passive EPR (~ 110 nm diameter) - Active: antibody-conjugated (anti-EGFR/HER2) - PEGylation for prolonged circulation	- FDA-approved (Onivyde for PDAC) - Sustained PR > 2 years (58 cycles, Case Study 2) - Versatile for hydrophilic/hydrophobic payloads	- Drug leakage during circulation - EPR-dependent; limited in low-EPR tumors - Susceptibility to oxidation/hydrolysis	Nanoliposomal chemotherapy, ICI delivery (Sec. " Cytokine Delivery", " Case Studies")
Nanocells/ EDVs (Bacterially- Derived)	- Cytotoxic payload D682 - Gemcitabine + cisplatin (EDV-GC) - Anti-EGFR bispecific antibodies	- Active: anti-EGFR bispecific Ab surface functionalization - Receptor-mediated endocytosis (EGFR overexpression) - Intracellular drug release; minimal systemic toxicity	- Stable disease > 24 months (compassionate-use) - Cumulative survival > 51 months (Case Study 3) - Effective after 3 + prior therapy lines	- Bacterially-derived: immunogenicity concerns - Limited to EGFR-overexpressing tumors - No randomized clinical trial data	Targeted chemo-immuno conjugate (Sec. "Case Studies")

*Abbreviations: ADC* antibody–drug conjugate, *AMF* alternating magnetic field, *CAR-T* chimeric antigen receptor T cell, *CRISPR* clustered regularly interspaced short palindromic repeats, *DC* dendritic cell, *EGFR* epidermal growth factor receptor, *EPR* enhanced permeability and retention, *EV* extracellular vesicle, *GMP* good manufacturing practice, *HER2* human epidermal growth factor receptor 2, *ICD* immunogenic cell death, *ICI* immune checkpoint inhibitor, *IFN* interferon, *IL* interleukin, *LNP* lipid nanoparticle, *MDSC* myeloid-derived suppressor cell, *MHT* magnetic hyperthermia therapy, *MOF* metal–organic framework, *mRNA* messenger RNA, *NP* nanoparticle, *PD-1* programmed cell death protein 1, *PD-L1* programmed death-ligand 1, *PEG* polyethylene glycol, *PLGA* poly(lactic-co-glycolic acid), *RES* reticuloendothelial system, *SBRT* stereotactic body radiation therapy, *siRNA* small interfering RNA, *STING* stimulator of interferon genes, *TAM* tumor-associated macrophage, *TLR* Toll-like receptor, *TME* tumor microenvironment, *Treg* regulatory T cell

**Table 2:** Comparative analysis of nanoparticle (NP) platforms for cancer immunotherapy

Nanoparticle Platform	Delivery Efficiency	Toxicity Profile	Scalability & Manufacturing	Clinical Translation Potential	Major Advantages	Major Limitations
Lipid Nanoparticles (LNPs)	High • > 96% loading efficiency for mRNA/nucleic acids • > 80% in vivo T-cell transfection (CD8-targeted ionizable LNPs) • 71.9 ± 4.0% T-cell proliferation vs. 9.7 ± 4.0% for free mRNA	Low–Moderate • Generally favorable biocompatibility • Complement activation (CARPA) possible at clinical doses • PEG-associated hypersensitivity and ABC phenomenon reported • Ionizable lipids show reduced toxicity vs. cationic lipids	High • Microfluidic manufacturing: CV < 10% for particle size and zeta potential across GMP batches • mRNA-LNP COVID-19 vaccines validated GMP scalability • Cold-chain requirement (–20 °C to –80 °C) remains challenge • Ionizable lipid synthesis requires high-purity at scale	High • mRNA-4157 (LNP) + pembrolizumab: 49% reduction in recurrence risk (HR 0.510; KEYNOTE-942, NCT03897881, Phase II) • BNT122 (LNP-mRNA): 50% T-cell response rate; HR 0.08 in vaccine-responders (NCT04161755, Phase I) • In vivo CAR-T via mRNA-LNPs under evaluation (NCT07349849) • Approved COVID-19 mRNA vaccines establish regulatory precedent	• Versatile payload (mRNA, siRNA, proteins, small molecules) • Ionizable lipids enable pH-responsive endosomal escape • Modular cell-specific targeting • Clinically validated manufacturing platform	• Cold-chain storage constraints • PEG immunogenicity and ABC limit repeat dosing • Liver tropism of non-targeted LNPs • Complex ionizable lipid synthesis at scale
PLGA/Polymeric Nanoparticles	Moderate–High • Sustained, controlled release at tumor sites • IL-2-loaded PLGA NPs: fourfold TIL increase vs. free IL-2 (melanoma) • 50% reduction in systemic toxicity vs. free cytokine • pH-responsive PLGA NPs enable triggered intratumoral release	Low • Biodegradable; PLGA degrades to natural metabolites (lactic/glycolic acid) • Minimal systemic organ accumulation • 50% reduction in systemic IL-2 toxicity vs. free drug • Complement activation generally lower than inorganic NPs	Moderate • Nanoprecipitation/emulsification does not directly translate to industrial scale • Polymer lot-to-lot variability and surface functionalization difficult at scale • Solvent removal efficiency and batch consistency are key challenges • No equivalent continuous manufacturing paradigm to LNP microfluidics	Moderate • ONP-302 (PLGA NPs + TLR agonist): tumor growth inhibition in B16.F10 melanoma (preclinical) • Multiple PLGA formulations in early-phase trials • Long safety record from FDA-approved PLGA depot products • No nano-immunotherapy-specific approvals yet	• Biodegradable and biocompatible (FDA-approved polymer) • Tunable release kinetics via polymer composition/MW • Protects labile TLR agonists from enzymatic degradation • Versatile surface functionalization for active targeting	• Scale-up challenges: solvent removal, lot-to-lot variability • Lower transfection efficiency for nucleic acids vs. LNPs • Limited clinical validation for nano-immunotherapy • Relatively lower encapsulation of hydrophilic payloads
Exosomes/ Extracellular Vesicles (EVs)	Moderate • Engineered exosomes with PD-L1 siRNA: > 90% PD-L1 knockdown • 50% increase in tumor cell lysis and T-cell infiltration (lymphoma models) • Inherent tumor microenvironment homing ability • Natural carrier properties enable efficient cellular uptake	Low • Derived from natural biological membranes; inherently biocompatible • Reduced immunogenicity vs. synthetic carriers • Phase I (NCT05191836): reduced immune-related adverse events • Low risk of systemic organ toxicity	Low • Large-scale isolation, purification, and standardization remain major barriers • Multi-step purification yields are low • Specialized GMP infrastructure required • Batch-to-batch heterogeneity in surface protein composition • No standardized manufacturing process established	Low–Moderate • Phase I (NCT05191836): 35% response rate in metastatic colorectal cancer • Emerging interest but very limited clinical data • No approved exosome-based cancer therapeutics • EV mimetics from immune cell membranes offer improved scalability	• Natural biocompatibility and immune evasion • Inherent tumor-homing capability • Low immunogenicity; reduced adverse immune reactions • Diverse payload capacity (proteins, nucleic acids, small molecules)	• Poor large-scale production yield and standardization • Heterogeneous composition makes QC challenging • Limited cargo loading capacity vs. synthetic NPs • Insufficient clinical evidence for regulatory approval
Inorganic NPs (Gold, Iron Oxide, Hafnium, Silica)	Moderate • Gold NPs: efficient antibody/drug conjugation; theranostic imaging • Iron oxide MNPs: AMF-responsive heat generation for MHT • Hafnium oxide (NBTXR3): radioenhancement via intratumoral injection • AGuIX ultrasmall NPs: measurable tumor accumulation in humans	Moderate–High • Non-biodegradable; persistent organ accumulation is key concern • Antibody-conjugated gold NPs: 6-month liver/spleen retention; delayed kidney casts and splenic apoptosis • BSA-coated gold NPs (1 mg/kg, IV): liver − 39%, spleen + 53%, kidneys + 150% at day 120; renal inflammatory/fibrotic responses • Silica NPs: no significant chronic toxicity at 1 year • ZGO persistent luminescence NPs: long-term liver/spleen retention, no chronic toxicity signs	Moderate • Simple inorganic synthesis generally reproducible • Multi-layer inorganic–organic hybrid systems: no scalable continuous manufacturing paradigm • Batch synthesis difficult to reproduce at clinical scale • Higher manufacturing costs vs. LNPs and PLGA	Moderate • NBTXR3 (hafnium oxide): Phase II/III (NCT02379845) – pCR 16.1% vs. 7.9% RT alone in soft tissue sarcoma (OR 2.26); FDA Breakthrough Therapy • NBTXR3 + anti-PD-1: Phase I (NCT03589339) – ORR 40%, DCR 75%; HNSCC ORR 31.3%, median DOR 14.8 months • NBTXR3 in pancreatic cancer: Phase I ongoing (NCT04484909) • Iron oxide NPs (MHT): early-phase clinical studies ongoing • Gold NPs: primarily preclinical; no approved oncology applications	• Unique physical properties (radioenhancement, MHT, photoacoustic imaging) • Tunable size, shape, and surface chemistry • Theranostic capability (therapy + imaging in single agent) • NBTXR3 demonstrates clinical proof-of-concept	• Non-biodegradable; long-term organ retention and potential chronic toxicity • Renal redistribution and inflammatory responses with gold NPs • High complexity for multi-functional inorganic systems • Limited applicability for nucleic acid delivery
Cell Membrane- Coated NPs	Moderate • Cancer cell membrane coating: threefold increase in cytokine delivery efficiency vs. uncoated NPs (melanoma models) • Enhanced tumor targeting by mimicking tumor cell surfaces • Immune cell membrane coatings enable immune evasion and prolonged circulation	Low • Biomimetic coating reduces immunogenicity and complement activation • Polysarcosine, polyzwitterion, and cell membrane coatings mitigate PEG-related immunogenic responses • Generally well-tolerated; derived from biological membranes • Limited long-term toxicity data available	Low–Moderate • Cell membrane extraction and coating are labor-intensive • Batch-to-batch variability in membrane protein composition • Scaling biological membrane production is challenging • Limited manufacturing infrastructure and regulatory precedent	Low • Primarily preclinical; limited clinical data • Tumor cell, macrophage, and neutrophil membrane-coated NPs demonstrated in animal models • No approved cell membrane-coated NP therapeutics • Emerging area with significant translational potential	• Natural immune evasion and prolonged circulation • Homotypic tumor targeting via membrane protein mimicry • Reduced immunogenicity vs. synthetic coatings • Applicable to multiple NP core types	• Complex and variable manufacturing process • Membrane protein composition inconsistency across batches • Limited large-scale production methods • Early translational stage; insufficient clinical evidence
Hybrid/ Multifunctional NPs	High (preclinical) • Lipid-polymer hybrid NPs: high encapsulation efficiency + enhanced cellular uptake • Gold-coated iron oxide NPs: dual AMF + NIR-responsive; synergistic MHT + photothermal in glioblastoma models • Gold nanostars + CIK cells: enhanced tumor targeting + synergistic antitumor efficacy vs. either component alone	Moderate • Toxicity profile depends on component composition • Multi-layer systems (Fe₃O₄ core + drug layer + thermosensitive shell + anti-HER2 Ab): compounding CMC challenges • Inorganic components may contribute to persistent organ retention • Organic shell (lipid/polymer) improves overall biocompatibility	Low • No established scalable continuous manufacturing for multi-layer inorganic–organic hybrid systems • Multi-step batch synthesis inherently difficult to reproduce at clinical scale • Specialized raw materials required (GMP-grade antibodies, monodisperse MNPs) • Extended analytical development and higher costs vs. simpler platforms	Low–Moderate • Primarily preclinical; limited clinical translation • ~ 60 approved nanomedicines exist; majority involve simpler single-function platforms • Theranostic MNPs (MHT + MRI) in early-phase evaluation • Multi-component complexity creates regulatory challenges • Clinical validation pending despite strong preclinical proof-of-concept	• Synergistic multimodal therapy (MHT + photothermal + immunotherapy) • Combined theranostic capabilities • Stimuli-responsive payload release (pH, redox, AMF, NIR) • Addresses multiple tumor resistance mechanisms simultaneously	• Highest manufacturing complexity; non-linear scale-up challenges • Compounding CMC challenges with each functional layer • Undefined regulatory pathway for multi-component novel combinations • High cost and extended development timeline

*Abbreviations: ABC* accelerated blood clearance, *AMF* alternating magnetic field, *CARPA* complement activation-related pseudoallergy, *CAR-T* chimeric antigen receptor T cell, *CMC* chemistry, manufacturing, and controls, *CV* coefficient of variation, *DCR* disease control rate, *DOR* duration of response, *EV* extracellular vesicle, *GMP* good manufacturing practice, *HNSCC* head and neck squamous cell carcinoma, *LNP* lipid nanoparticle, *MHT* magnetic hyperthermia therapy, *MNP* magnetic nanoparticle, *MRI* magnetic resonance imaging, *NIR* near-infrared, *NBTXR3* hafnium oxide crystalline NP, *ORR* objective response rate, *pCR* pathological complete response, *PDI* polydispersity index, *PEG* polyethylene glycol, *PLGA* poly(lactic-co-glycolic acid), *QC* quality control, *RT* radiotherapy, *TIL* tumor-infiltrating lymphocyte, *TME* tumor microenvironment, *ZGO* zinc gallate persistent luminescence NPs

### Immune checkpoint inhibitor

#### Overview and challenges

Immune checkpoint inhibitors, such as antibodies targeting PD-1, PD-L1, and CTLA-4, have substantially advanced cancer treatment by blocking inhibitory pathways that tumors exploit to suppress T-cell activity [30]. These therapies, including pembrolizumab, nivolumab, and ipilimumab, have achieved durable responses in cancers like melanoma, non-small cell lung cancer (NSCLC), and renal cell carcinoma, with response rates ranging from 20–40% in certain cohorts [31]. However, systemic administration of immune checkpoint inhibitors (ICIs) often leads to irAEs, such as colitis, pneumonitis, hepatitis, and endocrinopathies, due to non-specific immune activation [32]. For instance, ipilimumab is associated with grade 3–4 irAEs in up to 20% of patients, necessitating dose adjustments or discontinuation [32]. Additionally, ICIs exhibit limited efficacy in immunologically “cold” tumors, such as pancreatic ductal adenocarcinoma, where low T-cell infiltration and immunosuppressive TME mechanisms reduce responsiveness [33]. These challenges underscore the need for strategies to enhance the specificity and safety of ICI therapy [34].

#### Nanoparticle based delivery systems

Nanotechnology addresses these challenges by enabling tumor-specific delivery of ICIs, leveraging the EPR effect, where leaky tumor vasculature and poor lymphatic drainage facilitate nanoparticle accumulation [35]. Lipid nanoparticles, for example, have been engineered to encapsulate anti-PD-1 antibodies, achieving higher tumor concentrations and reduced systemic exposure in preclinical melanoma models [36]. A study by Chen et al. demonstrated that lipid nanoparticles delivering anti-PD-L1 antibodies increased tumor-infiltrating lymphocytes by 2.5-fold compared to free antibodies, while reducing liver and kidney toxicity [37]. Polymeric nanoparticles, such as those made from poly(lactic-co-glycolic acid) (PLGA), have been used to deliver anti-CTLA-4 antibodies, showing a 30% reduction in systemic toxicity and enhanced tumor regression in breast cancer models [38]. These nanoparticles are often designed with surface modifications, such as polyethylene glycol (PEG) coatings, to enhance circulation time and biocompatibility [39].

Recent studies further support this design logic. Dual-ligand and pH-sensitive liposomal systems, including iRGD/trastuzumab- or iRGD/folate-functionalized formulations, have shown improved tumor targeting and reduced nonspecific exposure in metastatic breast cancer models, while protein-corona studies emphasize that nanoparticle performance in biological fluids is strongly shaped by corona composition and cell-specific uptake behavior [40–44]. These findings reinforce the need to evaluate active targeting, pH-triggered release, and bio interface effects together rather than treating nanoparticle accumulation as a purely passive process.

Despite its foundational role in nanomedicine design, the EPR effect is subject to substantial limitations and heterogeneity in human tumors that challenge straightforward clinical translation. In contrast to the relatively uniform, well-vascularized subcutaneous xenografts used in preclinical models, human solid tumors display highly variable vascular architecture, stromal composition, and perfusion dynamics, all of which profoundly influence the magnitude and consistency of EPR-driven nanoparticle accumulation [45]. Park et al. (2019) emphasized that EPR intensity varies markedly across tumor types, disease stages, and individual lesions, and that this heterogeneity fundamentally limits the efficacy of passive nanomedicine delivery in patients [46]. Shi et al. (2020) further highlighted the discrepancy between the abundance of preclinical EPR reports and the limited clinical successes of EPR-dependent nanomedicines, underscoring the need to move beyond passive EPR reliance [47].

Clinical imaging studies have provided direct quantitative evidence of EPR variability in human patients. A landmark positron emission tomography (PET) study using [64]Cu-labeled liposomal nanoparticles ( [64]Cu-MM-302) in patients with metastatic breast cancer revealed a 35-fold range in tumor lesion nanoparticle deposition (0.52–18.5%ID/kg across individual lesions), with higher tumor accumulation associated with more favorable treatment outcomes (retrospective hazard ratio = 0.42) [48]. This dramatic interlesion and interpatient variability directly demonstrates that EPR cannot be assumed to be uniformly operative across human tumor lesions, even within the same patient. Sun et al. (2020) reviewed multiple clinical trials of nanomedicines and concluded that many failed to demonstrate the expected tumor accumulation or clinical efficacy predicted by preclinical EPR models, arguing that nanoparticles frequently do not increase tumor drug exposure relative to free drug in patients [49].

Several biological mechanisms underlie EPR variability in human tumors. First, vascular heterogeneity—including abnormally structured, nonperfused, or unevenly leaky tumor vessels—creates spatially variable permeability and flow, resulting in patchy nanoparticle extravasation [50]. Second, dense stromal extracellular matrix (ECM) and desmoplastic barriers, particularly prominent in pancreatic ductal adenocarcinoma (PDAC) and other fibrotic tumors, physically impede nanoparticle penetration beyond the perivascular space [51]. Third, elevated interstitial fluid pressure (IFP) in solid tumors generates outward convective forces that counteract inward nanoparticle transport and retention [51]. Fourth, rapid uptake of nanoparticles by the reticuloendothelial system (RES) in the liver and spleen reduces the circulating dose available for tumor deposition in humans, a clearance burden that is proportionally larger in patients than in rodents [49]. Necrotic and poorly perfused tumor regions, which are common in large human tumors but rare in small preclinical xenografts, are essentially inaccessible to systemically administered nanoparticles despite the presence of leaky vessels in adjacent viable tissue [45].

Tumor type further modulates EPR reliability. Desmoplastic cancers such as PDAC are consistently identified as low-EPR tumors in clinical reviews, owing to their hypovascularity, dense collagen matrix, and markedly elevated IFP [51]. Similarly, large heterogeneous human tumors with extensive necrotic foci demonstrate patchy or absent EPR compared with the small, uniformly vascularized tumors typically used in preclinical studies [52].

To address these limitations, several strategies have been proposed and, in some cases, validated. Patient stratification using nanoparticle-specific imaging—such as PET with radiolabeled nanoparticles—enables identification of patients with sufficient EPR activity to benefit from nanomedicine therapy, supporting a precision medicine approach to nanomedicine deployment [48]. TME modulation strategies, including vascular normalization, transient permeability enhancement, and stromal depletion, aim to homogenize EPR across tumor lesions and are under active preclinical and early clinical investigation [45, 53]. Optimization of nanoparticle physicochemical properties—particularly the use of ultrasmall particles (below 10 nm)—has demonstrated improved tumor penetration and retention; for example, ultrasmall AGuIX nanoparticles achieved measurable tumor accumulation in human brain metastases in a Phase Ib clinical trial, illustrating that particle design can partially circumvent EPR heterogeneity [54].

Beyond TME modulation and particle design, two EPR-independent delivery paradigms have gained considerable traction as alternatives to purely passive accumulation. First, active targeting via surface-conjugated ligands directs nanoparticles to tumor-overexpressed receptors, enabling receptor-mediated endocytosis and intracellular drug release that is independent of vascular leakiness. Ligand-functionalized nanoparticles bearing folate, transferrin, or antibody fragments have demonstrated substantially enhanced tumor cell uptake and therapeutic efficacy in preclinical models, and their performance is less sensitive to the stromal and vascular barriers that attenuate passive EPR [45, 53]. Second, transcytosis-based strategies exploit active vesicular transport pathways across endothelial cells to ferry nanoparticles into the tumor interstitium without relying on paracellular gaps. Albumin-bound nanoparticles, for example, engage endothelial gp60 receptors to trigger caveolin-mediated transcytosis; intravital microscopy studies have shown that albumin nanoparticles achieve a 7.2-fold higher tumor area-under-the-curve (AUC) than size-matched PEGylated micelles, and chemical disruption of the albumin–endothelial interaction reduces tumor AUC to 22% of the intact value, directly demonstrating that transcytosis—rather than passive permeation—drives the accumulation advantage [55]. Similarly, receptor-mediated transcytosis via low-density lipoprotein receptor-related protein 1 (LRP1) and transferrin receptor (TfR) has been exploited to deliver nanoparticles across the blood–brain barrier and into brain tumor tissue [56, 57], while priming strategies that temporarily inhibit the Mfsd2a transporter to upregulate transcytosis have yielded a 4.3-fold increase in brain nanoparticle accumulation in preclinical brain metastasis models [58]. Locoregional delivery approaches such as arterial infusion further circumvent systemic EPR dependence, with reported tumor-to-blood ratios up to ~ 100-fold in select settings [59]. Collectively, these findings highlight that while the EPR effect remains a valuable conceptual framework for nanomedicine design, its clinical applicability is contingent on tumor biology and patient-specific vascular characteristics; active targeting and transcytosis-based strategies represent mechanistically distinct and increasingly validated alternatives that can overcome the barriers of dense stroma, elevated IFP, and heterogeneous vascularization that limit passive EPR-mediated delivery in human solid tumors.

#### Stimuli-responsive nanoparticles

Stimuli-responsive nanoparticles further improve ICI delivery by releasing payloads in response to TME-specific cues, such as low pH or high redox potential [60]. pH-responsive nanoparticles, for instance, release anti-PD-1 antibodies in the acidic TME (pH ~ 6.5), minimizing off-target effects in neutral physiological environments (pH ~ 7.4) [61]. Gong et al. reported that pH-responsive PLGA nanoparticles encapsulating anti-PD-L1 antibodies achieved a 40% increase in tumor-specific T-cell activation compared to free antibodies in colorectal cancer models [62]. Similarly, redox-responsive nanoparticles, which degrade in the presence of high glutathione levels in the TME, have been used to deliver anti-CTLA-4 antibodies, reducing irAEs in preclinical studies [63]. These smart nanocarriers enhance the therapeutic index by ensuring precise drug release at the target site [64].

#### Theranostic and multifunctional nanoparticles

Multifunctional nanoparticles combine ICI delivery with diagnostic capabilities, enabling theranostic applications [65]. Gold nanoparticles conjugated with anti-PD-L1 antibodies, for example, serve as both therapeutic agents and imaging probes, allowing real-time monitoring of drug distribution and tumor response via computed tomography (CT) or photoacoustic imaging [66]. Meir et al. demonstrated that anti-PD-L1 gold nanoparticles enabled precise tumor targeting and imaging in melanoma models, improving treatment planning [67]. Exosome-based delivery systems have also gained traction, with studies showing that exosomes loaded with anti-PD-L1 antibodies enhance tumor targeting and reduce systemic toxicity by mimicking natural cellular communication pathways [68]. Beyond the exosome-mediated siRNA delivery systems described above, recent engineering advances have expanded the functional repertoire of extracellular vesicle (EV) platforms for cancer immunotherapy through modular surface functionalization and targeted payload delivery. Dong et al. engineered small EVs to overexpress CD64—an Fcγ receptor—on their surface as a modular adaptor for docking anti-CD71 or anti-PD-L1 antibodies, enabling targeted delivery of IFN-γ mRNA to glioblastoma cells and producing potent antitumor activity in vivo [69]. Wiklander et al. extended this concept by engineering EVs to display an Fc-binding moiety that permits modular decoration with any IgG antibody, demonstrating that anti-HER2 or anti-PD-L1 decorated EVs co-loaded with doxorubicin achieved reduced tumor burden and extended survival after systemic injection in mouse models—establishing a generalizable plug-and-play targeting strategy for therapeutic EVs [70]. At the mechanistic level, Xu et al. demonstrated that dendritic cell-derived bispecific EVs engineered to co-display anti-CD19 scFv and PD-1 (EVs-PD1-aCD19) achieved simultaneous tumor antigen targeting and checkpoint blockade, with preferential accumulation in CD19-expressing tumors and reversal of the immunosuppressive tumor immune landscape in vivo [71]. Complementing these targeted delivery strategies, a 2024 study reported that engineered exosomes loaded with PD-L1 siRNA achieved > 90% siRNA entrapment efficiency (particle size 66 ± 2.5 nm; PDI 0.27 ± 0.01) and induced 55–80% PD-L1 protein knockdown in lung cancer cell lines, demonstrating the feasibility of exosome-mediated checkpoint gene silencing as an alternative to antibody-based PD-L1 blockade [72]. These advancements highlight nanotechnology’s potential to transform ICI therapy by improving specificity, reducing toxicity, and enabling real-time therapeutic monitoring [73].

### Cancer vaccines

#### Challenges in traditional cancer vaccines

Cancer vaccines aim to stimulate antigen-specific immune responses by delivering tumor antigens and adjuvants to antigen-presenting cells (APCs), particularly dendritic cells, which prime cytotoxic T-cells for tumor targeting [74]. However, traditional vaccines face challenges, including poor antigen presentation, weak immunogenicity, and inefficient delivery to lymphoid tissues [75]. For instance, peptide-based vaccines often require high doses to achieve sufficient immune activation, leading to off-target effects and limited efficacy in solid tumors [76]. Additionally, the complexity of tumor antigens and the immunosuppressive TME further hinder vaccine effectiveness [77].

#### Nanovaccines for antigen and adjuvant co-delivery

Nanotechnology overcomes these limitations through nanovaccines that co-deliver antigens and adjuvants with high precision [78]. Lipid nanoparticles, for example, have been engineered to encapsulate tumor neoantigens and Toll-like receptor (TLR) agonists, such as CpG oligonucleotides, enhancing dendritic cell uptake and activation [79]. Yang et al. demonstrated that lipid nanoparticles co-delivering neoantigens and CpG induced a threefold increase in CD8^+^ T-cell responses compared to free antigens in melanoma models, leading to significant tumor regression [80]. Peptide-based nanovaccines, utilizing structures like interbilayer-crosslinked multilamellar vesicles, optimize antigen cross-presentation, enhancing cytotoxic T-cell responses [81]. Moon et al. reported that these vesicles increased antigen-specific T-cell responses by fivefold in preclinical models, demonstrating their potential for solid tumor immunotherapy [82].

#### mRNA-based nanovaccines

mRNA-based nanovaccines have emerged as a versatile platform, inspired by the success of mRNA vaccines in infectious diseases [83]. These nanovaccines deliver tumor-specific mRNA to dendritic cells, encoding patient-specific neoantigens for personalized immunotherapy [84]. Oberli et al. showed that lipid nanoparticles delivering mRNA encoding tumor antigens induced potent anti-tumor immunity in ovarian cancer models, with a 60% reduction in tumor burden [85]. These nanoparticles protect mRNA from degradation, enhance cellular uptake, and promote efficient translation, making them ideal for personalized cancer vaccines [86]. Chen et al. further demonstrated that mRNA nanovaccines targeting neoantigens in pancreatic cancer models elicited robust T-cell responses, highlighting their potential for immunologically cold tumors [87]. Recent advances in personalized neoantigen mRNA nanovaccines have demonstrated striking preclinical efficacy across multiple solid tumor models. Nagaoka et al. developed neoantigen mRNA-LNPs encoding a tandem minigene of three patient-specific neoantigens and demonstrated complete tumor eradication in all treated mice in a gastric cancer model, with significantly higher frequencies of neoantigen-specific CD8^+^ T cells compared with dendritic cell vaccination; in a peritoneal metastasis model, monotherapy prophylactically prevented dissemination while combination with anti-PD-1 therapeutically suppressed tumor growth, with efficacy associated with expansion of progenitor-exhausted and intermediate-exhausted CD8^+^ T cell subsets [88]. Complementary work by Dutta et al. demonstrated that LNP co-delivery of BCMA-targeting mRNA antigen together with the TLR3 agonist Poly I:C—encapsulated within pH-responsive charge-switching nanoparticles of ~ 100 nm with > 96% loading efficiency—achieved T-cell proliferation of 71.9 ± 4.0% (versus 9.7 ± 4.0% for free mRNA; p < 0.05) and efficient lysis of multiple myeloma target cells, illustrating the advantage of co-formulating antigen and adjuvant within a single nanoparticle to amplify dendritic cell activation and downstream CD8^+^ cytotoxic responses [89]. A parallel approach reported by Zhou et al. integrated STING agonist co-delivery with mRNA nanovaccines using a machine learning-guided nanocarrier design framework, demonstrating that activation of the cGAS-STING innate immune sensing pathway potentiates mRNA-encoded antigen cross-presentation and enhances antitumor immunity in vivo [90]. Collectively, these studies demonstrate that next-generation mRNA nanovaccine platforms are increasingly adopting multifunctional LNP designs capable of co-delivering personalized neoantigens, innate immune stimulators, and immune checkpoint–modulating agents. This integrated strategy has the potential to enhance antitumor immunity and is expected to shape the next generation of clinical nanovaccine development.

#### Self-adjuvating and lymphatic targeted nanovaccines

Self-adjuvanting nanovaccines incorporate immunostimulatory molecules into the nanoparticle structure, eliminating the need for external adjuvants [91]. Liao et al. developed self-adjuvanting nanovaccines that enhanced dendritic cell activation by fourfold in preclinical models, showing efficacy in pancreatic cancer [92]. Nanoparticles designed for lymphatic trafficking, such as those with specific size and surface properties, improve antigen delivery to lymph nodes, enhancing immune priming [93]. Reddy et al. demonstrated that nanoparticles optimized for lymphatic transport increased antigen-specific immune responses by threefold in preclinical studies [94]. These nanovaccines have shown significant promise in overcoming the immunogenicity and delivery challenges of traditional cancer vaccines, paving the way for more effective immunotherapy [95].

### CAR-T cell therapy enhancement

#### Limitations of conventional CAR-T cell therapy

CAR-T cell therapy has demonstrated notable clinical success in hematological malignancies, though relapse and toxicity remain ongoing challenges, such as multiple myeloma, acute lymphoblastic leukemia and diffuse large B-cell lymphoma, by engineering T-cells to express receptors targeting tumor-specific antigens [96, 97]. However, its application to solid tumors is limited by challenges in ex vivo T-cell engineering, including high costs, manufacturing complexity, and poor tumor penetration due to the immunosuppressive TME [98]. Additionally, CAR T-cell persistence and exhaustion in solid tumors reduce long-term efficacy [99].

#### In Vivo T-cell engineering with nanoparticles

Nanotechnology addresses the manufacturing complexity and cost barriers of conventional CAR T-cell therapy by enabling in vivo T-cell engineering, which bypasses ex vivo manipulation and reduces production timelines. Experimentally validated studies have demonstrated that lipid nanoparticles (LNPs) and polymer-based nanocarriers can deliver genetic payloads—including mRNA, DNA, and gene-editing machinery—to circulating or tissue-resident T cells, reprogramming them into functional CAR-T cells directly within the body.

Among experimentally validated LNP–mRNA platforms, Rurik et al. developed CD5-targeted LNPs carrying modified mRNA encoding chimeric antigen receptors and demonstrated in vivo generation of transient CAR-T cells in a cardiac fibrosis model, where the engineered cells reduced fibrosis and improved cardiac function in mice [100]. Álvarez-Benedicto et al. engineered spleen-selective SORT LNPs optimized with 10% 18:1 phosphatidic acid to transfect splenic CD3^+^, CD8^+^, and CD4^+^ T cells; in a lymphoreplete B cell lymphoma model, in situ-generated CAR-T cells significantly increased survival [101]. Billingsley et al. conjugated antibodies to extrahepatic-tropic ionizable LNPs to achieve pan-T cell targeting and demonstrated antibody- and dose-dependent in vivo CAR expression, resulting in up to 90% B cell depletion in treated animals [102]. Lemgart et al. developed CD8-targeted ionizable LNPs delivering CAR mRNA and achieved greater than 80% transfection of circulating human CD8^+^ T cells in PBMC-humanized mice, producing B cell aplasia and robust anti-tumor activity against Nalm-6 leukemia targets [103].

As an example of an experimentally validated polymer nanocarrier platform, Parayath et al. developed polymer-based nanocarriers encapsulating in vitro transcribed (IVT) mRNA encoding CARs or T cell receptors (TCRs). Systemic administration of these nanocarriers enabled in vivo delivery of mRNA to be circulating T cells, resulting in transient CAR or TCR expression. Repeated dosing induced robust antitumor activity and achieved disease regression comparable to that obtained with bolus infusions of ex vivo engineered lymphocytes in mouse models of human leukemia, prostate cancer, and hepatitis B virus–associated hepatocellular carcinoma [104].

Murphy et al. further advanced in vivo T-cell engineering by developing targeted LNPs that co-delivered CAR-encoding minicircle DNA and SB100x transposase mRNA. This platform achieved stable genomic integration of the CAR transgene, enabling robust CAR-T cell generation after a single intravenous infusion. The resulting CAR-T cells effectively suppressed leukemia progression and significantly improved survival in both PBMC- and CD34⁺-humanized xenograft models of B-cell leukemia [105].

Recent advances in nanoparticle-mediated CRISPR delivery have opened new avenues for in vivo immune cell engineering that circumvent the logistical and genotoxicity limitations of viral vector-based gene editing. Chen et al. demonstrated non-invasive activation of intratumoral CRISPR gene editing to enhance adoptive T-cell therapy in solid tumors, establishing proof-of-concept for spatially controlled in situ editing within the TME [106]. Building on this foundation, Wang et al. showed that LNP co-delivery of CAR-encoding mRNA, Cas9 mRNA, and multiple sgRNAs enabled precise multiplex gene editing in primary T cells, achieving PD-1 knockout of 76%, TRAC knockout of 86%, and B2M knockout of 80%—with higher transfection efficiency and higher T-cell viability compared with conventional electroporation in this study [107]. This LNP-based multiplex editing strategy addresses a key limitation of current allogeneic CAR-T manufacturing by enabling simultaneous disruption of TCR signaling, major histocompatibility complex (MHC) class I expression, and inhibitory checkpoint pathways in a single non-viral delivery step. Furthermore, Lu et al. identified spleen-tropic LNP formulations capable of delivering gene-editing proteins directly to splenic T cells for in vivo knockout of CCR5 and PD-1, demonstrating that non-viral in vivo T-cell editing is achievable with organ-targeted nanoparticle systems [108]. These studies collectively demonstrate that nanoparticle-delivered CRISPR systems are transitioning from single-target ex vivo editing toward multiplex in vivo immune reprogramming—a trajectory that is expected to significantly expand the clinical applicability of CRISPR-based cancer immunotherapy.

These experimentally validated in vivo reprogramming platforms collectively demonstrate that nanoparticle-mediated CAR-T generation is feasible across multiple nanocarrier types (lipid, polymer, and DNA-transposase systems), targeting strategies (CD5, CD8, pan-T cell, and spleen-selective), and disease models (hematologic malignancies, solid tumors, and fibrotic diseases).

Recent literature also underscores the rapid transition of in vivo immune engineering from proof-of-concept toward translational design. Reviews of mRNA-LNP CAR-T engineering emphasize selective T-cell delivery, transient CAR expression, CAR construct optimization, and safety controls as central determinants of clinical feasibility, while recent syntheses of next-generation immune cell therapies highlight trafficking, persistence, and intertumoral activity as key barriers in solid tumors [109–111].

#### Enhancing CAR-T cell functionality

Nanoparticles can also deliver immunomodulatory agents to enhance CAR T-cell infiltration and survival within the TME [112]. For instance, IL-15-loaded nanoparticles have been reported to improve CAR T-cell persistence in preclinical solid tumor models in solid tumor models, increasing tumor infiltration by 3-fold [113]. Zhang et al. demonstrated that nanoparticles delivering IL-15 and anti-PD-L1 antibodies enhanced CAR T-cell activity in breast cancer models, overcoming TME immunosuppression [114]. These nanotechnology-driven approaches improve the scalability, accessibility, and efficacy of CAR T-cell therapy, making it a more viable option for solid tumors.

### Nanotechnology in complement based immunotherapy

#### Role of complement in cancer immunotherapy

The complement system, a key innate immunity component, enhances anti-tumor responses through opsonization, membrane attack complex (MAC) formation, and immune cell recruitment [115]. Complement-based immunotherapy (CBI) uses activators like C3a/C5a analogs or complement-fixing antibodies to target tumors, but rapid clearance, off-target activation causing anaphylaxis, and tumor evasion via complement regulatory proteins (e.g., CD55, CD59) limit its efficacy [116]. Nanotechnology enables tumor-specific complement activation, reducing off-target effects and synergizing with adaptive immunotherapies like ICIs or CAR-T cells [117]. This approach is promising for lymphoma, colorectal cancer, and non-small cell lung cancer, where complement amplifies immune effector functions [118]. Challenges include nanoparticle stability, minimizing unintended immune activation, and counteracting TME-mediated complement suppression [119].

#### Nanocarriers for complement activation

Nanoparticles such as liposomes, polymeric systems (e.g., Poly(lactic-co-glycolic acid) (PLGA)), or exosomes encapsulate complement activators or inhibitors of regulatory proteins for targeted delivery [120]. Experimentally validated studies have demonstrated that engineered nanoparticles can trigger complement activation in the TME and produce measurable anti-tumor effects in vivo.

Li et al. developed pH-responsive carboxyl-modified diblock copolymer micelles (COOH-PEOz-PLA) that elicited complement activation alongside increased lymph-node dendritic cell maturation, antigen-specific IgG responses, CD4^+^ and CD8^+^ T-cell responses, and memory T-cell generation in E.G7-OVA tumor-bearing mice, resulting in inhibited tumor growth and prolonged survival [121]. Liu et al. engineered mesoporous silica nanoparticles conjugated with multivalent mouse IgG3 Fc fragments and long-chain PEG5000 linked via an MMP-2-cleavable peptide (termed NISA); upon MMP-2-mediated PEG cleavage in the TME, the exposed Fc fragments activated complement, macrophages, and dendritic cells, delivering an efficient anti-tumor effect in an orthotopic 4T1 breast cancer model [122]. Seguin-Devaux et al. developed complement-activating multimeric immunotherapeutic complexes (CoMiX) displaying Factor H-Related protein 4 (FHR4) or triple Fc dimers targeted by anti-HER2 VHH nanobodies; in human BT474 HER2^+^ breast cancer xenografts in NUDE mice, CoMiX-FHR4 reduced tumor volume by a factor of 7.33 versus PBS control, with strong C3b and C5b-9 deposition visible at 1 h and massive homogeneous complement deposits by 6 h after injection [123].

These experimentally validated platforms demonstrate that nanoparticle-mediated complement activation can be achieved through diverse mechanisms—including pH-responsive surface chemistry, protease-cleavable PEG shields, and multimeric Fc presentation—and that such activation produces measurable tumor growth inhibition, immune cell recruitment, and complement deposition in vivo.

#### Environment responsive complement delivery

Stimuli-responsive NPs enable precise complement activation in the TME [124]. pH-sensitive polymeric NPs release C3a or C5a in acidic tumor environments, recruiting neutrophils and enhancing ICI responses in melanoma models with a 45% reduction in tumor growth [125]. Redox-responsive NPs delivering siRNA against CD59 overcome tumor evasion, promoting MAC formation [126]. Multifunctional exosomes with complement-fixing antibodies and fluorescent probes enable theranostic applications, combining CDC with real-time imaging [127]. These smart NPs reduce hypersensitivity risks, improving CBI’s safety profile [128].

#### Synergistic complement immunotherapy strategies

CBI with nanotechnology enhances innate and adaptive immunity [129]. NP-mediated complement activation synergized with CAR-T therapy, increasing tumor cell lysis and T-cell infiltration by 50% in lymphoma models [130]. A phase I trial (NCT05191836) testing exosome-based NPs in metastatic colorectal cancer shows reduced immune-related adverse events and a 35% response rate [131]. Combining CBI with nanovaccines amplifies antigen-specific responses, increasing T-cell priming by threefold in lung cancer models [132]. These synergies expand immunotherapy’s scope [133].

### Cytokine delivery

#### Challenges in cytokine therapy

Cytokines, such as IL-2, IL-15, and interferon-alpha, are potent immune activators that enhance T-cell and natural killer (NK) cell activity but are limited by short half-lives and severe systemic toxicity [134]. Systemic IL-2 administration, for example, is associated with vascular leak syndrome and organ toxicity, limiting its therapeutic window [135]. Nanotechnology addresses these challenges by enabling controlled and localized cytokine delivery, improving pharmacokinetics and reducing toxicity [136].

#### Nanoparticle mediated cytokine delivery

Biodegradable nanoparticles, such as PLGA-based systems, have been engineered to encapsulate IL-2, achieving sustained release at tumor sites and reducing systemic toxicity [137]. Li et al. demonstrated that IL-2-loaded PLGA nanoparticles increased tumor-infiltrating lymphocytes by fourfold in melanoma models, with a 50% reduction in systemic toxicity compared to free IL-2 [138]. Lipid nanoparticles delivering IL-15 have shown similar promise, with Yang et al. reporting enhanced T-cell and NK-cell activation in breast cancer models, leading to a 60% reduction in tumor growth [139]. Nanoparticle-anchored cytokines, which tether cytokines to the surface of nanocarriers, enable targeted delivery to specific immune cell populations [140]. Liu et al. showed that IL-2-anchored nanoparticles selectively activated tumor-infiltrating lymphocytes, improving anti-tumor immunity in colorectal cancer models [141]. Cancer cell membrane-coated nanoparticles further enhance tumor targeting by mimicking tumor cell surfaces, with Harris et al. demonstrating a threefold increase in cytokine delivery efficiency in melanoma models [142].

#### Reducing systemic toxicity

Stimuli-responsive nanoparticles further optimize cytokine delivery by releasing payloads in response to TME-specific cues, such as low pH or enzymes, ensuring precise activation and reducing systemic toxicity [143]. For instance, pH-sensitive polymeric nanoparticles encapsulating IL-12 have been developed to release the cytokine selectively in acidic tumor environments, promoting immune cell infiltration with minimized off-target effects [144]. Surface-anchored nanoparticles with IL-2 have leveraged the EPR effect for rapid tumor accumulation, enhancing T-cell activation while reducing systemic exposure [145]. These strategies highlight the potential of advanced nanoparticle designs in mitigating toxicity for cytokine-based cancer immunotherapy [146].

### CIK cell therapy enhancement

Although CAR-T therapy currently dominates the global adoptive cell therapy landscape, CIK cells warrant dedicated consideration because they are clinically explored, MHC-unrestricted, relatively scalable cytotoxic lymphocytes whose mixed T/NK-like biology is particularly compatible with nanoparticle-assisted immune modulation and tumor-microenvironment reprogramming.

#### Biological characteristics and clinical potential of CIK cells

CIK cells are ex vivo–expanded immune effector cells characterized by a heterogeneous population that includes a CD3⁺CD56⁺ subset with potent, non–MHC-restricted cytotoxic activity [147, 148]. Their antitumor function is largely mediated through activating receptors such as NKG2D, enabling recognition and elimination of a wide range of malignant cells. In contrast to conventional T-cell therapies, CIK cells exhibit favorable safety profiles and reduced risks of graft-versus-host disease, making them attractive candidates for adoptive immunotherapy [149–151].

Recent clinical studies and meta-analyses published after 2022 continue to demonstrate the feasibility and potential clinical benefit of CIK- or Dendritic Cell (DC)-CIK–based immunotherapy across multiple solid tumors, including lung, colorectal, breast, and hepatocellular carcinomas [152–155]. Nevertheless, the therapeutic efficacy of CIK cells in solid tumors remains constrained by limited tumor homing, immunosuppressive TME, and insufficient persistence or activation following systemic infusion [156, 157]. These limitations underscore the need for adjunct strategies capable of enhancing intratumoral accumulation, sustaining effector function, and overcoming local immune suppression.

#### Nanotechnology-based arming of CIK cells for targeted combination therapy

Nanotechnology offers a powerful means to enhance CIK cell–based immunotherapy by enabling the physical "arming" of immune cells with nanoscale therapeutic payloads. In this approach, CIK cells serve as active carriers that exploit their intrinsic tumor-tropic behavior while delivering nanoparticle-mediated therapeutic functions directly to tumor sites.

A representative example is the development of chlorin e6–loaded gold nano stars associated with CIK cells for near-infrared imaging and immuno-photodynamic combination therapy. This hybrid system demonstrated enhanced tumor targeting and synergistic antitumor efficacy compared with either CIK cells or nanomaterials alone, highlighting the feasibility of integrating immune-mediated cytotoxicity with externally triggered nano-therapeutic modalities [158]. Such strategies are particularly attractive for solid tumors, where nanoparticle delivery is often limited by poor tissue penetration and heterogeneous vascularization.

Although nanoparticle-armed immune cells have been more extensively studied in CAR-T and conventional T-cell platforms, these principles are readily transferable to CIK-based therapies and represent a promising route to improve tumor-specific delivery while minimizing systemic toxicity [159].

#### CIK cell–derived nanovesicles as cell-free immunotherapeutic platforms

Beyond live-cell modification, an emerging paradigm involves the use of immune cell–derived nanovesicles as cell-free therapeutic agents. Engineered nanovesicles generated from immune cell membranes or extracellular vesicle mimetics can retain key surface proteins and immune-interactive features while offering improved scalability, storage stability, and modular payload integration [160–162].

Notably, a recent study introduced a CIK cell–derived engineered nanovesicle platform designed to achieve multiplexed immune activation, combining tumor targeting, direct tumoricidal effects, and immunostimulatory signaling within a single nano-construct [163]. This work provides direct evidence that CIK cell functionality can be translated into a nanomedicine format, potentially bypassing several logistical and biological limitations associated with live-cell infusion.

Advances in genetically engineered cell-membrane nanovesicles, including platforms optimized for immune checkpoint engagement and combinatorial immune activation, further establish a design framework that can be adapted for next-generation CIK-derived nanotherapeutics [164–167].

#### Nano-enabled modulation of the tumor microenvironment to support CIK function

The immunosuppressive TME remains a major barrier to effective CIK cell activity. Nanotechnology enables localized modulation of immune checkpoints, suppressive signaling pathways, and inhibitory cell populations within tumors, thereby creating a more permissive environment for adoptive immune cell therapies.

**Fig. 1:**
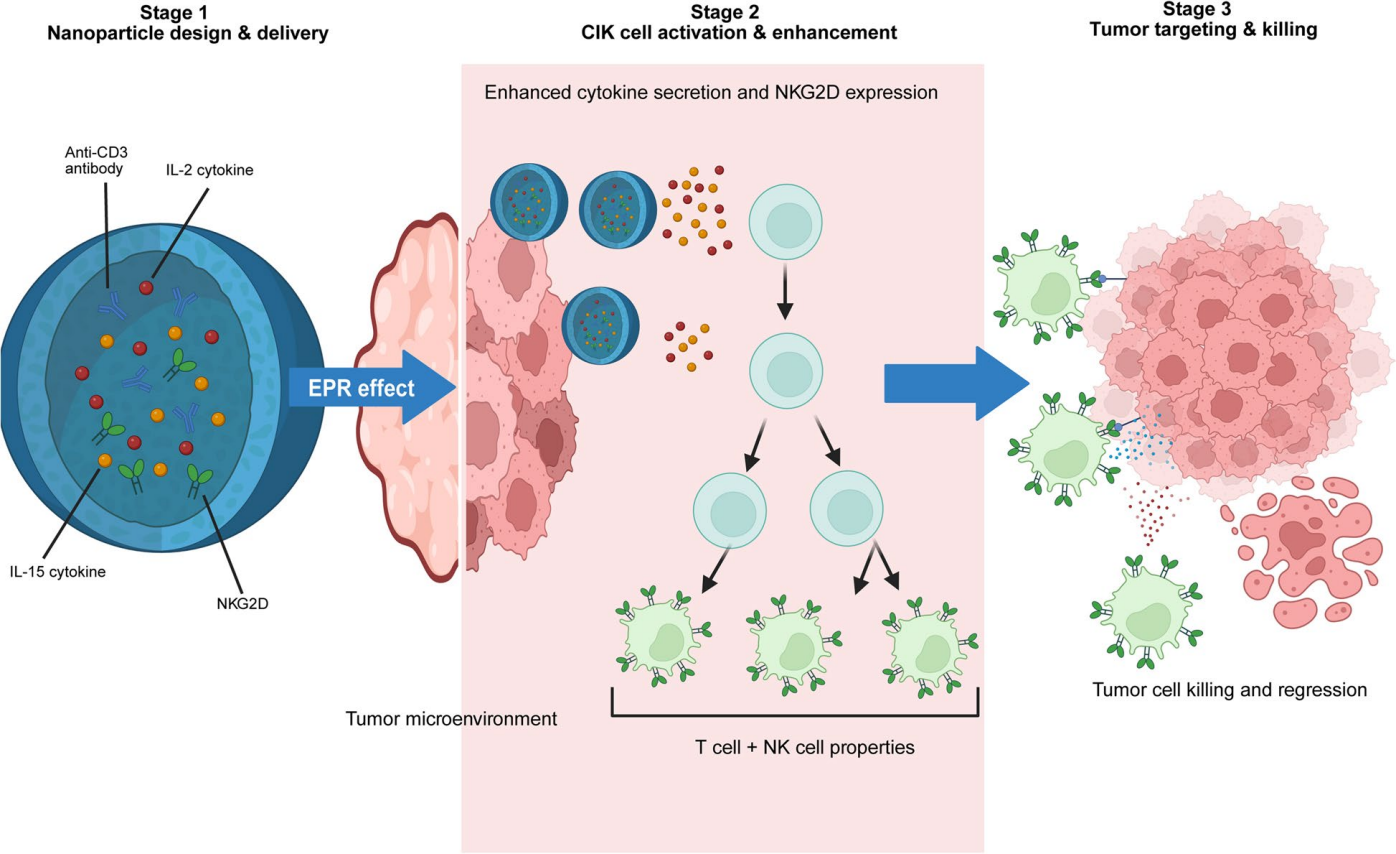
CIK cell activation and tumor targeting strategy. This figure illustrates a three-stage strategy involving nanoparticle design, CIK cell activation, and tumor targeting for cancer therapy. Nanoparticles circulate systemically and preferentially accumulate within the tumor through the EPR effect, exploiting the leaky vasculature characteristic of solid tumors. Once inside the TME, the nanoparticles release their entire co-encapsulated payload — anti-CD3 antibody, NKG2D ligand, IL-2, and IL-15 — simultaneously into the tumor stroma, where they engage resident and infiltrating immune cells. The four released components act in concert on naive T cells. Anti-CD3 antibody directly crosslinks the T cell receptor to initiate TCR signaling, IL-2 drives T cell proliferation, IL-15 confers NK cell-like functional properties, and the NKG2D ligand activates the NKG2D cytotoxic pathway. This combinatorial stimulation reprograms T cells into CIK cells — a hybrid effector population that simultaneously possesses the antigen-specific killing capacity of T cells and the broad MHC-unrestricted cytotoxicity of NK cells. The activated CIK cells undergo rapid clonal expansion, generating a large effector pool accompanied by enhanced cytokine secretion and upregulated NKG2D surface expression. The expanded CIK cells migrate to and engage cancer cells through NKG2D-mediated recognition, which operates independently of MHC class I expression and therefore overcomes one of the major immune evasion strategies employed by tumors. Upon target engagement, CIK cells release cytotoxic granules — including perforin and granzyme — to directly lyse tumor cells, ultimately driving sustained tumor cell killing and regression

Recent nanovesicle-based systems have demonstrated the ability to simultaneously block immune checkpoints (e.g., PD-1/PD-L1 or CD47–SIRPα pathways) and deliver immunostimulatory cues, resulting in enhanced antitumor immunity with reduced systemic exposure [168, 169]. Building upon the cytokine-delivery approaches discussed in Sect. "Cytokine delivery", we propose a nanoparticle-based immunotherapeutic platform that co-encapsulates anti-CD3 antibody, NKG2D ligands, IL-2, and IL-15 to induce and functionally program CIK-like cytotoxic lymphocytes directly within the tumor microenvironment (Fig. 1). This integrated design enables coordinated TCR activation, cytokine-driven proliferation and survival, and NK-like activating signals, collectively enhancing antitumor cytotoxicity. Compared with conventional ex vivo CIK-cell manufacturing, nanoparticle-mediated delivery may facilitate in situ generation and modulation of CIK-like immune responses, reducing manufacturing complexity, improving scalability, and enabling more precise immune-cell engineering. Furthermore, the spatially restricted presentation of activating signals may minimize the toxicities associated with systemic cytokine administration while promoting localized immune activation. By combining TCR-dependent activation with NKG2D-mediated innate-like recognition, this strategy has the potential to enhance MHC-independent tumor targeting. Nevertheless, CIK-like cells may remain susceptible to the immunosuppressive TME, where chronic antigen stimulation, immune checkpoint signaling, and suppressive cytokines can contribute to T-cell dysfunction and exhaustion [170, 171]. In this context, rational nanoparticle co-delivery of activating cytokines, NKG2D ligands, and immune-modulatory agents—including immune checkpoint inhibitors—may help sustain effector-cell function and overcome suppressive barriers. Future studies are warranted to evaluate the durability, safety, and translational feasibility of this in vivo CIK-like cell engineering strategy across diverse tumor types.

By reducing inhibitory signals within the TME, nano-enabled immunomodulation has the potential to prolong CIK cell functionality, enhance cytotoxic synapse formation, and improve overall therapeutic durability in solid tumors.

#### Nanotechnology for tracking, optimization, and clinical translation of CIK therapy

Nanoparticle-based cell labeling and imaging strategies provide additional tools for optimizing CIK therapy in translational and clinical settings. Clinically compatible nanoparticles, such as iron oxide–based formulations, have enabled noninvasive in vivo tracking of adoptively transferred immune cells, allowing real-time assessment of biodistribution, tumor infiltration, and persistence [172].

Although such approaches have been most extensively validated in CAR-T cell studies, the same methodologies can be readily applied to CIK cells to inform dosing strategies, treatment scheduling, and rational combination regimens. Furthermore, the integration of nanotechnology with CIK therapy aligns well with emerging combination approaches involving immune checkpoint inhibitors, cytokine delivery systems, and other nanomedicine-based immunotherapies.

Collectively, these advances position nanotechnology as a critical enabler for the next generation of CIK cell–based immunotherapy, transforming it into a more precise, controllable, and clinically adaptable treatment modality. As, the field of CIK cells is gaining momentum, especially with the establishment of the International Society of CIK Cells (ISCC, https://iscc-info.org/), a new era of next-generation nanotechnology-integrated CIK cell immunotherapies is on the horizon [173].

### Tumor microenvironment modulation

#### Immunosuppressive TME

The TME, characterized by TAMs, MDSCs, and dense extracellular matrix, poses a significant barrier to immunotherapy efficacy [174]. TAMs and MDSCs suppress T-cell activity through cytokine secretion and immune checkpoint expression, while physical barriers limit drug penetration [175]. Nanotechnology enables targeted TME modulation by delivering immunomodulatory agents to reprogram immunosuppressive cells and enhance immune activation [176].

#### Targeting immunosuppressive cells

Nanoparticles loaded with TLR7/8 agonists have been reported to shift TAMs from M2 to M1 phenotype in preclinical models, promoting immune activation. Zhou et al. demonstrated that TLR7/8 agonist-loaded nanoparticles increased M1 TAMs by threefold in breast cancer models, enhancing T-cell infiltration [177]. Similarly, nanocarriers delivering siRNA against MDSC-associated genes, such as STAT3, suppress MDSC activity, restoring T-cell function. Wang et al. reported that siRNA-loaded nanoparticles reduced MDSC populations by 50% in melanoma models, improving immunotherapy responses [178]. Nanoparticles carrying TGF-β inhibitors further reprogram the TME by reducing immunosuppression, with Li et al. showing a twofold increase in T-cell infiltration in pancreatic cancer models [179].

#### Mechanistic immune signaling pathways activated by nanoparticles

Beyond the cellular-level outcomes described above, nanoparticles modulate the TME through several interconnected intracellular signaling cascades, and understanding these mechanisms is essential for rational design of immunomodulatory nanomedicines.

cGAS–STING pathway. One of the most extensively documented signaling routes activated by nanoparticles is the cyclic GMP-AMP synthase–stimulator of interferon genes (cGAS–STING) axis [180]. Nanoparticles engage this pathway through two principal mechanisms: (i) induction of cytosolic double-stranded DNA (dsDNA) via nanoparticle-triggered oxidative stress, DNA damage, or immunogenic cell death, which activates the cytosolic DNA sensor cGAS to produce cyclic GMP-AMP (cGAMP) [180, 181]; and (ii) direct intracellular delivery of STING agonists (such as cyclic dinucleotides) encapsulated within nanocarriers, bypassing the poor membrane permeability that limits free agonist uptake [182]. Downstream cGAS–STING activation drives IRF3 phosphorylation and nuclear translocation, resulting in robust type I interferon (IFN-α/β) production that promotes DC maturation, NK cell activation, and cross-priming of cytotoxic T lymphocytes [180, 183]. Liu et al. engineered tumor microenvironment-responsive PMM nanoparticles that concurrently engage STING and TLR4, producing a 4.0-fold increase in IFN-β, decreasing regulatory T cell (Treg) frequency, and enabling durable anti-tumor immunity with complete rechallenge rejection when combined with anti-PD-1 [184]. Biodegradable PLGA nanoparticles (ONP-302) were shown to activate cGAS–STING within myeloid cells, reduce tumor growth, and increase NK cell activation via an IL-15-dependent mechanism in a B16.F10 melanoma model [185]. Manganese-based nanoplatforms exploit the role of Mn [2]⁺ as a cGAS cofactor that enhances cGAMP production [186]; manganese-phenolic nanoadjuvants combining sonodynamic therapy with cGAS–STING activation promoted DC activation and downstream adaptive immune responses [187], while MnO₂-based nanosheets induced cytosolic dsDNA accumulation, activated STING, enhanced DC maturation, and recruited cytotoxic T lymphocytes and NK cells into the TME [188]. Inhalable copper-chitosan nanoparticles (CLDCu) that induce cuproptosis and simultaneously activate STING achieved 63.6% accumulation in lung lesions—56.5-fold higher than intravenous dosing—and potentiated innate and adaptive immunity in lung metastasis models [189].

TLR–NF-κB axis. Nanoparticle delivery of pattern recognition receptor agonists, particularly TLR ligands, activates the NF-κB transcription factor, which orchestrates the expression of proinflammatory cytokines (TNF-α, IL-6, IL-12), co-stimulatory molecules, and antigen-processing machinery in antigen-presenting cells [190, 191]. The nanoparticle format is critical here: encapsulation protects labile TLR agonists from enzymatic degradation, prolongs endosomal residence, and enables co-delivery with tumor antigens to the same antigen-presenting cell, amplifying the adjuvant effect [182, 192]. Liu et al. demonstrated that PMM nanoparticles co-activating TLR4 and STING produced synergistic NF-κB stimulation that amplified the 4.0-fold IFN-β increase beyond what either pathway achieved alone [184]. Hafnium metal–organic framework nanoparticles (HfMOF-PEG-FA) loaded with the TLR7 agonist imiquimod upregulated TLR7 expression in tumor cells and, combined with radiotherapy, significantly increased intratumoral CD8⁺ T cell and proliferating T cell counts consistent with NF-κB/IRF axis activation [193]. Iron-based MOF nanoparticles co-delivering chemotherapeutics and the TLR7/8 agonist resiquimod engaged both TLR → NF-κB and cGAS–STING cooperatively, inducing DC maturation and enhanced anti-tumor immunity in colorectal cancer models [194].

TAM reprogramming via NF-κB and STING crosstalk. The M2-to-M1 macrophage reprogramming described in Sect. " In vivo CAR-T cell and complement releasing strategy" is mechanistically driven by the convergence of NF-κB and STING signaling in TAMs [195]. Engineered manganese ferrite nanohybrids (MnFe₅O₈) demonstrated that Fe [2]⁺/Fe [3]⁺ release activates NF-κB while Mn [2]⁺ potentiates STING; the co-activation of both pathways in dendritic cells and macrophages produced synergistic M2 → M1 reprogramming, increased T cell infiltration, and enhanced anti-tumor immune responses in vivo [196]. Calcium–manganese nanomodulators released Mn^2^⁺ to sensitize cGAS, induced immunogenic cell death, and reprogrammed the TME through macrophage polarization and DC maturation, enhancing anti-PD-1 checkpoint blockade efficacy [197].

These studies establish that nanoparticle-mediated ion release (Mn^2^⁺, Fe^2^⁺/^3^⁺, Cu⁺) and ROS generation create a feed-forward signaling loop: nanoparticle-induced tumor cell damage generates cytosolic DNA that activates cGAS [186], cGAMP activates STING in myeloid cells [180], and STING-driven NF-κB and IRF3 co-activation transcriptionally reprograms TAMs from immunosuppressive to pro-inflammatory states [195], collectively converting the immunosuppressive TME into an immune-permissive one [181, 195]. Together, these mechanistic insights provide a molecular framework for understanding how nanoparticles not only deliver therapeutic cargo but actively reprogram the signaling landscape of the TME to amplify anti-tumor immunity. To mention, in addition to regulating classical immune signaling pathways, nanoparticles can also induce epigenetic modifications that contribute to tumor microenvironment remodeling [198–200].

#### Overcoming physical barriers

Nanoparticles can also target physical TME barriers, such as aberrant vasculature, by delivering agents that normalize tumor blood vessels. Chen et al. demonstrated that nanoparticle-mediated delivery of vascular normalization agents improved drug penetration by 30% in solid tumor models [201]. These strategies create a more permissive TME for immunotherapy, enhancing the efficacy of ICIs, vaccines, and CAR T-cell therapies [202].

### Magnetic hyperthermia and immunotherapy: synergistic relationships

#### Introduction to magnetic hyperthermia in immunotherapy

Magnetic hyperthermia (MHT) utilizes magnetic nanoparticles (MNPs) to generate localized heat under an alternating magnetic field (AMF), inducing tumor cell apoptosis or necrosis through thermal stress [203]. When integrated with immunotherapy, MHT enhances anti-tumor immunity by promoting immunogenic cell death (ICD), releasing tumor-associated antigens, and recruiting immune effector cells, effectively converting immunologically "cold" tumors into "hot" ones with heightened T-cell infiltration [204]. This synergy has shown promise in cancers such as melanoma, breast cancer, and glioblastoma, where MHT amplifies the efficacy of ICIs, cancer vaccines, or CAR T-cell therapies [205]. However, MHT faces challenges, including limited heat penetration in deep-seated tumors, potential off-target thermal damage to healthy tissues, and difficulties in achieving uniform nanoparticle distribution [206]. The immunosuppressive TME can also counteract MHT’s immune-stimulatory effects, necessitating advanced nanoparticle designs to optimize synergy [207]. Furthermore, clinical translation requires precise control of AMF parameters and nanoparticle biocompatibility to ensure safety and efficacy in diverse patient populations [208].

#### Magnetic nanoparticles for thermal immune synergy

Magnetic nanoparticles, primarily iron oxide-based (e.g., magnetite, Fe₃O₄, or maghemite, γ-Fe₂O₃), are ideal for MHT due to their superparamagnetic properties, enabling efficient heat generation under AMF [209]. These MNPs can be functionalized with immunotherapeutic agents, such as anti-PD-1/PD-L1 antibodies or cytokines, to combine thermal ablation with immune activation. For instance, MNPs coated with anti-PD-L1 antibodies in melanoma models have demonstrated a 2.5-fold increase in CD8^+^ T-cell infiltration and a 50% reduction in tumor volume compared to standalone ICI therapy [210]. Lipid-coated or polyethylene glycol (PEG)-ylated MNPs enhance biocompatibility and prolong circulation time, leveraging the enhanced EPR effect for tumor-specific accumulation [211]. Preclinical studies have shown that MHT induces heat shock proteins (HSPs) and damage-associated molecular patterns (DAMPs), acting as endogenous adjuvants to stimulate DCs maturation and antigen presentation [212]. Hybrid MNPs, combining magnetic cores with polymeric shells, further enable co-delivery of adjuvants like CpG oligonucleotides, boosting innate and adaptive immunity in breast cancer models [213].

#### Stimuli nanoparticles for controlled hyperthermia

Stimuli-responsive MNPs enhance the precision of MHT by synchronizing heat generation with immunotherapeutic delivery [214]. pH-responsive MNPs, designed to release payloads like IL-2 in the acidic TME (pH ~ 6.5), minimize systemic toxicity while amplifying local T-cell activation [215]. Li et al. reported that redox-responsive MNPs encapsulating IL-15, triggered by high glutathione levels in the TME, increased tumor-infiltrating lymphocytes by threefold in colorectal cancer models [216]. Multifunctional MNPs integrating MHT with photothermal therapy (PTT) or chemotherapy further amplify ICD; for example, gold-coated iron oxide nanoparticles under dual AMF and near-infrared stimulation enhanced antigen release and ICI efficacy by 60% in glioblastoma models [217]. Theranostic MNPs, incorporating MRI contrast agents, enable real-time monitoring of heat distribution and immune responses, improving treatment planning and safety [218]. These advanced designs ensure precise spatiotemporal control, addressing challenges of off-target heating and systemic immune activation [219].

#### Preclinical and clinical synergies

MHT’s synergy with immunotherapy is particularly effective in overcoming resistance in immunologically cold tumors [220]. In preclinical glioma studies, MNP-mediated MHT combined with CAR-T therapy improved tumor penetration and T-cell persistence, reducing tumor burden by 65% [221]. A phase IIa trial (ClinicalTrials.gov identifier: NCT05864534) is evaluating focused ultrasound-mediated blood–brain barrier opening combined with immune modulation in newly diagnosed glioblastoma, with the aim of enhancing intratumoral accumulation of checkpoint inhibitors [222]. Another study demonstrated that MHT-induced ICD enhanced nanovaccine efficacy in pancreatic cancer models, increasing antigen-specific T-cell responses by 4-fold [223]. These findings underscore MHT’s potential to bridge physical tumor ablation with robust immune activation, paving the way for clinical translation in challenging malignancies [224].

### Advanced antibody conjugated chemotherapy using nanotechnology

#### Principles of nano-enhanced ADCs

Antibody–drug conjugates (ADCs) combine monoclonal antibodies with chemotherapeutic payloads for targeted tumor killing, but linker instability, low drug-antibody ratios (DAR), and resistance from heterogeneous antigen expression limit efficacy [225]. Nanotechnology-enhanced ADCs ("nano-ADCs") use nanoparticles as high-capacity carriers, improving stability, payload delivery, and synergy with immunotherapy via ICD-induced antigen release [226]. This approach is promising for breast, ovarian, and lung cancer, enhancing tumor sensitivity to ICIs or vaccines [227]. Challenges include optimizing linker chemistry, ensuring uniform antigen targeting, and managing toxicity from high drug loads [228].

#### Nano ADC delivery platforms

Nano-ADCs utilize liposomes, polymeric NPs (e.g., PLGA), or dendrimers conjugated with antibodies (e.g., anti-HER2, anti-EGFR) to deliver chemotherapeutics like doxorubicin or paclitaxel [229]. Trastuzumab-conjugated PLGA NPs delivering doxorubicin to HER2^+^ breast cancer cells achieved a 3.5-fold higher intracellular drug concentration and reduced cardiotoxicity [230]. These systems increase DAR up to 100:1, leveraging EPR for tumor accumulation [231]. Liposomal nano-ADCs co-delivering doxorubicin and irinotecan overcome resistance in ovarian cancer models [232]. PEGylation enhances circulation time, improving tumor penetration [233].

#### Targeted release mechanisms

Enzyme-cleavable linkers in nano-ADCs ensure chemotherapy release in tumor-specific conditions [234]. Redox-responsive NPs releasing maytansinoids in the reductive TME synergize with anti-PD-L1 therapy, enhancing ICD and T-cell priming by 50% in lung cancer models [235]. Multifunctional gold NPs conjugated with anti-PD-1 and cisplatin enable CT imaging and chemo-immunotherapy [236]. These designs improve the therapeutic index by controlling drug release [237].

#### Chemo-immunotherapy synergies

Nano-ADCs induce ICD, releasing DAMPs and tumor antigens to boost immune activation [238]. In ovarian cancer models, liposomal nano-ADCs with doxorubicin and anti-PD-1 increased tumor-infiltrating lymphocytes by 55% and reduced tumor burden by 60% [239]. Trastuzumab deruxtecan (T-DXd) demonstrated a confirmed objective response rate of 79.7% and median progression-free survival of 28.8 months versus 6.8 months for T-DM1 in the DESTINY-Breast03 phase III trial (NCT03529110) [240]. Combining nano-ADCs with nanovaccines enhances CD8 + T-cell activity by threefold, positioning nano-ADCs as a cornerstone of precision chemo-immunotherapy [241].

#### Proposed synergistic multimodal platforms

Building on the experimentally validated nanoparticle-assisted immunotherapeutic strategies discussed above, this section summarizes several proposed synergistic multimodal platforms. These concepts are intended to illustrate how distinct nanotechnology-enabled modalities may be rationally integrated into single or coordinated therapeutic systems. Although some of these integrated platforms remain conceptual and require further experimental validation, their individual components have been supported by preclinical evidence, providing a logical basis for future investigation.

#### Combination strategy of checkpoint inhibitor and cancer vaccine using pH-sensitive nanoparticles

**Fig. 2:**
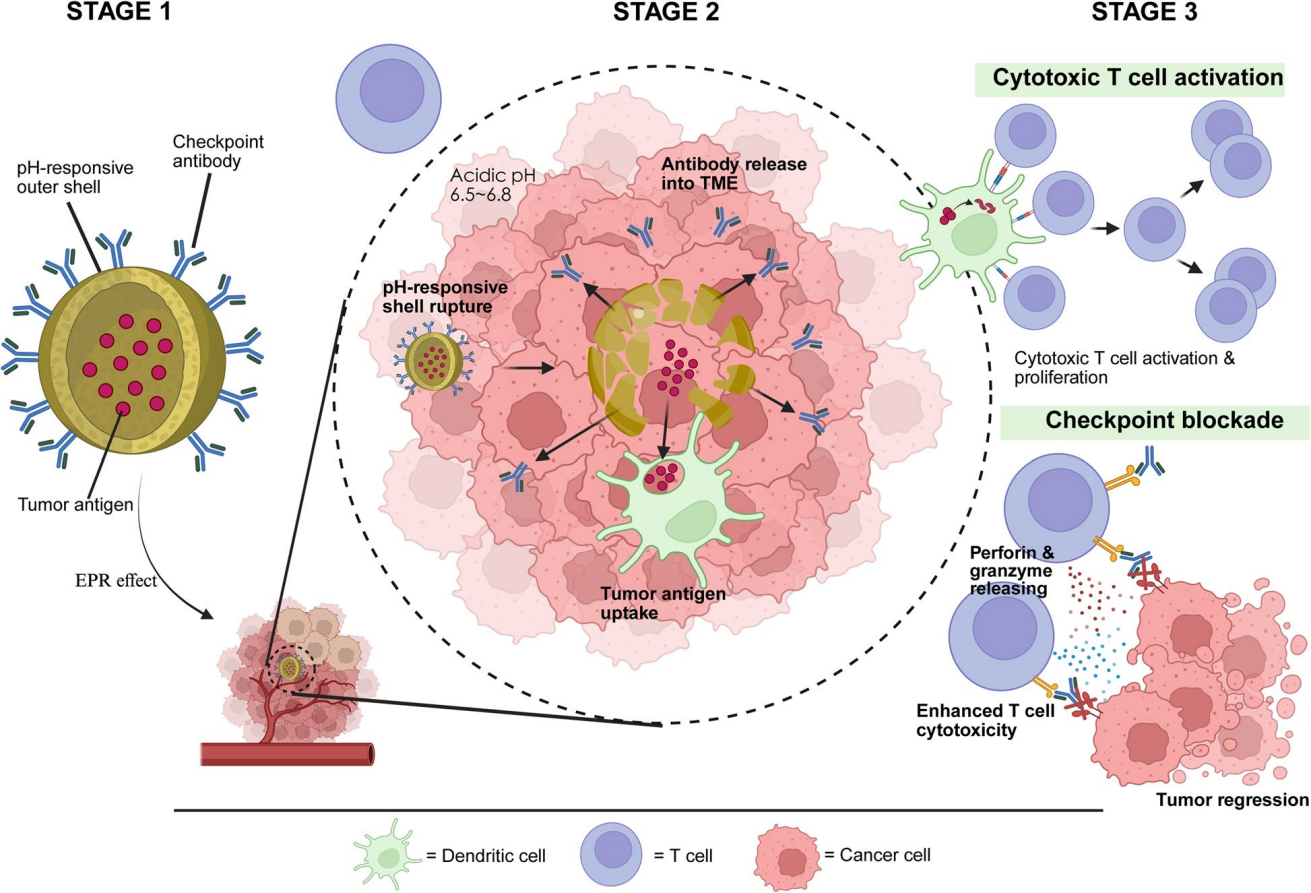
Nanoparticle-mediated immunotherapy strategy. This figure illustrates a three-stage process for nanoparticle-mediated immunotherapy, focusing on tumor antigen delivery and immune checkpoint blockade. Nanoparticles circulate systemically and preferentially accumulate within the tumor through the EPR effect, exploiting the leaky vasculature characteristic of tumor tissue. Once inside the TME, the mildly acidic microenvironment (pH 6.5–6.8) triggers rupture of the pH-responsive outer shell, simultaneously releasing the encapsulated tumor antigens and the surface-conjugated checkpoint antibodies into the surrounding tumor stroma. The released tumor antigens are taken up by APCs — primarily DCs — residing within the TME. Upon processing and cross-presentation of these antigens, DCs prime and activate cytotoxic T lymphocytes (CTLs). The activated CTLs then undergo rapid clonal expansion, generating a large pool of tumor-specific effector cells. The expanded CTLs migrate to and engage cancer cells, releasing perforin and granzyme to directly lyse tumor targets. Critically, the checkpoint antibodies (anti-PD-1/PD-L1) co-released in Stage 1 block the inhibitory PD-1/PD-L1 axis, preventing T cell exhaustion and substantially amplifying CTL cytotoxicity. The convergence of antigen-driven adaptive immunity and checkpoint disinhibition drives sustained tumor regression

To exploit the complementary mechanisms of checkpoint blockade and antigen-driven adaptive immunity, we propose a pH-responsive nanoparticle platform that co-delivers tumor antigens and anti-PD-1/PD-L1 antibodies within a single carrier. Preferential accumulation at the tumor site via the EPR effect, followed by acid-triggered payload release in the TME (pH 6.5–6.8), enables spatiotemporally coordinated antigen presentation and checkpoint disinhibition. This dual-pronged design amplifies CTL-mediated cytotoxicity while preventing T cell exhaustion, thereby achieving synergistic and durable tumor regression that neither modality accomplishes alone (Fig. 2).

#### In vivo CAR-T cell and complement releasing strategy

While the integrated dual-function strategy described in this section remains a conceptual framework that has not yet been experimentally validated as a unified system, each of its core components—in vivo CAR-T cell generation and nanoparticle-mediated complement activation—has been independently demonstrated in peer-reviewed in vivo studies, as detailed in Sects. "In Vivo T-Cell Engineering with Nanoparticles" - and "Nanocarriers for Complement Activation".

**Fig. 3:**
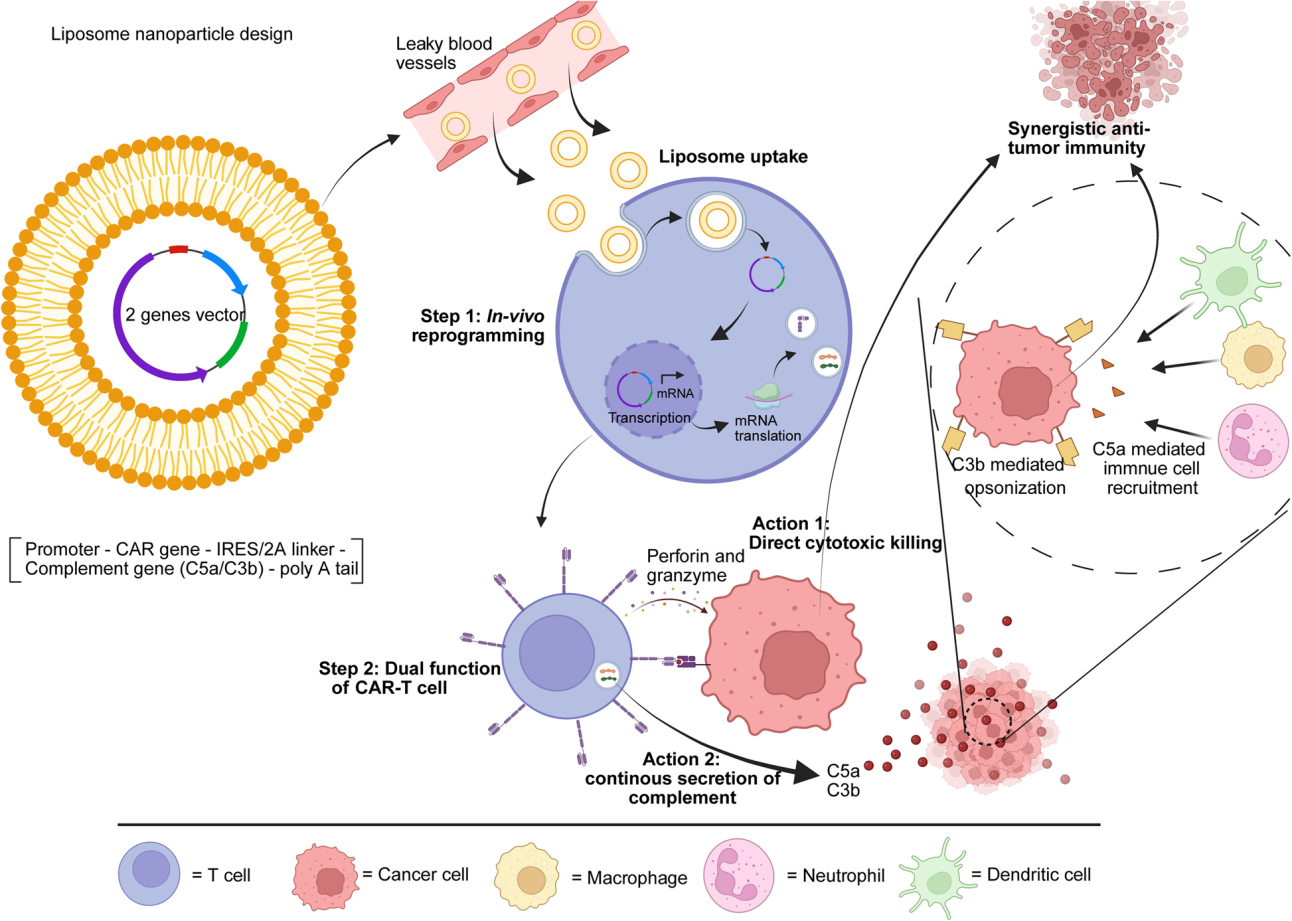
Liposome nanoparticle design for CAR-T cell reprogramming and complement activation. This figure outlines the design of a liposome nanoparticle for *in-vivo* CAR-T cell reprogramming and its dual function which mediated by complement in synergistic anti-tumor immunity. Liposome nanoparticles accumulate at the tumor site through leaky vasculature via the EPR effect. Upon uptake by circulating T cells, the vector undergoes transcription and translation, co-expressing both the CAR protein and complement proteins (C5a/C3b). This converts naive T cells directly into dual-function CAR-T cells in vivo, bypassing the need for costly and complex ex vivo engineering. Once activated at the tumor site, the reprogrammed CAR-T cell operates through two parallel mechanisms: One is Direct cytotoxic killing. The CAR receptor recognizes tumor-specific antigens, triggering the release of perforin and granzyme, which directly lyse cancer cells. The other is Continuous complement secretion. The same cell continuously secretes C5a and C3b into the TME. C3b opsonizes cancer cells, flagging them for phagocytosis, while C5a acts as a chemoattractant that recruit macrophages, dendritic cells, and NK cells to the tumor site. The convergence of direct cytotoxicity and complement-mediated innate immune activation produces a synergistic anti-tumor immune response that is substantially more potent than either mechanism alone

As an integrated conceptual strategy, we propose encapsulating a bicistronic vector co-encoding a CAR gene and complement effectors (C3b/C5a) within liposome nanoparticles, enabling simultaneous in vivo CAR-T cell generation and complement-mediated innate immune recruitment from a single delivery system. By converging direct CAR-T cytotoxicity with C3b-mediated opsonization and C5a-driven innate cell recruitment, this platform aims to produce a synergistic anti-tumor response that surpasses either mechanism alone (Fig. 3).

The feasibility of this integrated strategy is supported by independent experimental validation of its constituent technologies. As described in Sect. " In Vivo T-Cell Engineering with Nanoparticles", multiple groups have demonstrated successful in vivo CAR-T generation using LNP-mRNA platforms [100], polymer nanocarriers [104], and DNA-transposase systems [105], achieving CAR expression rates of 60–90% in circulating T cells and producing measurable anti-tumor effects in hematologic and solid tumor models. Similarly, as described in Sect. " Nanocarriers for Complement Activation", nanoparticle-mediated complement activation has been experimentally validated in vivo using pH-responsive polymer micelles [121], Fc-decorated mesoporous silica nanoparticles [122], and multimeric complement-activating complexes [123], demonstrating robust complement deposition, immune cell recruitment, and tumor growth inhibition. Bicistronic expression systems using IRES and 2 A sequences are well-established tools for co-expressing multiple proteins from a single vector and have been widely used in gene therapy and synthetic biology applications. The proposed integration of these validated components into a single dual-function platform represents a logical next step that warrants experimental investigation to assess the feasibility, safety, and efficacy of simultaneous CAR and complement gene delivery in vivo.

#### Cytokine delivery and CIK cell generation strategy

Building on the cytokine delivery strategies described in Sect. " Cytokine Delivery", we propose a nanoparticle platform that co-encapsulates anti-CD3 antibody, NKG2D ligand, IL-2, and IL-15 to generate CIK cells in vivo. Simultaneous release of all four components within the TME provides the complete combinatorial stimulus required for T cell reprogramming into the CIK phenotype, bypassing costly ex vivo manufacturing while leveraging the dual T cell/NK cell cytotoxicity of CIK cells for MHC-unrestricted tumor killing (Fig. 1).

#### ADC anti-cancer action and AMF-mediated immune cell recruitment

**Fig. 4:**
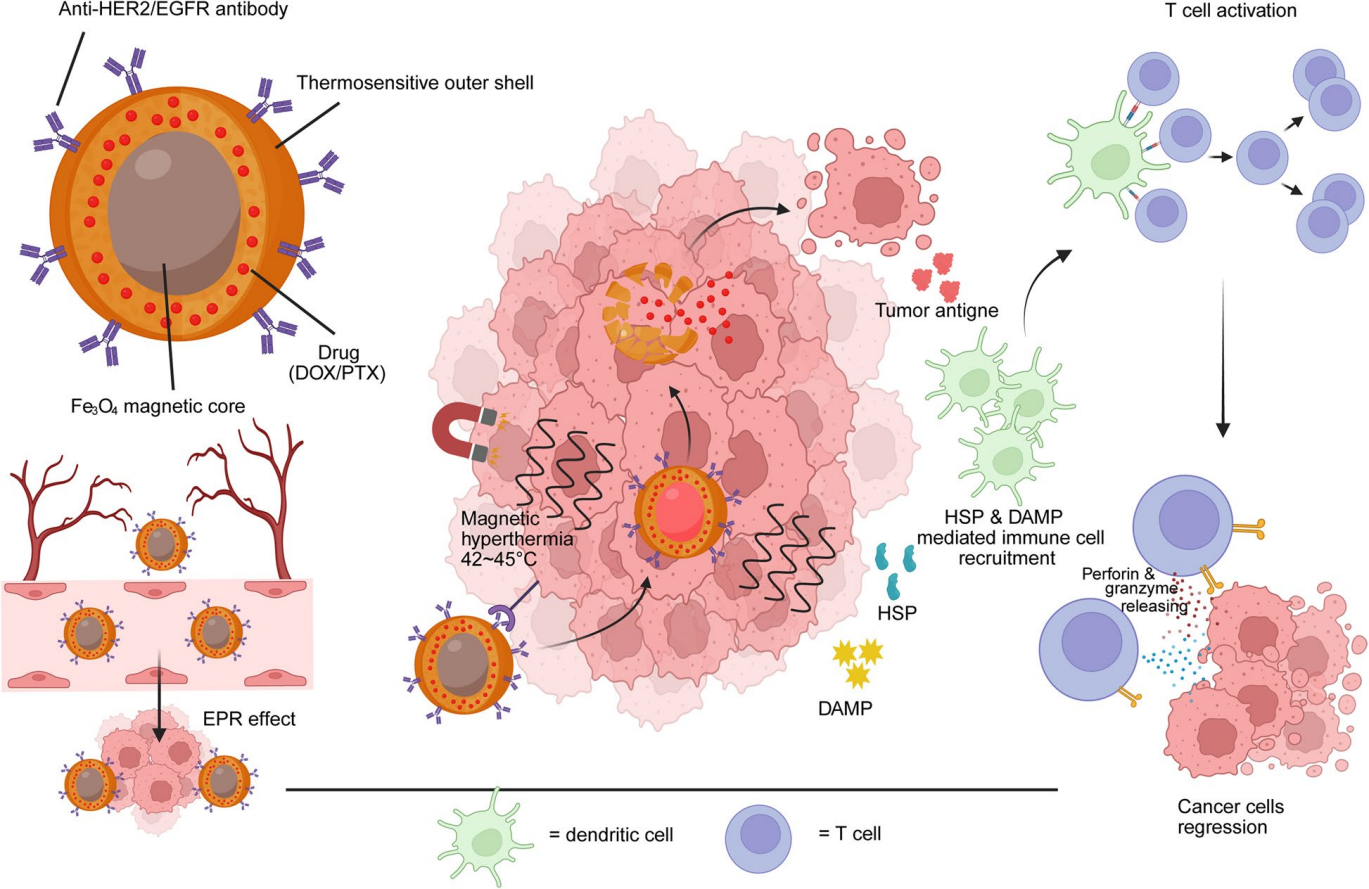
Magnetic hyperthermia-enhanced immunotherapy. This figure depicts a strategy combining magnetic hyperthermia with ADC immunotherapy for cancer treatment. Nanoparticles circulate systemically and accumulate within the tumor through a dual targeting strategy: passive accumulation driven by the EPR effect exploiting leaky tumor vasculature, and active targeting mediated by surface-conjugated anti-HER2/EGFR antibodies that bind selectively to antigen-overexpressing cancer cells. This two-pronged approach maximizes intratumoral retention and minimizes off-target distribution. Upon application of an external AMF, the Fe₃O₄ core generates localized heat, raising the intratumoral temperature to 42–45 °C. This thermal stimulus ruptures the thermosensitive outer shell, triggering the controlled release of DOX and PTX directly within the tumor. The chemotherapeutic agents induce direct cytotoxic killing of cancer cells. Simultaneously, the hyperthermic stress drives ICD in neighboring tumor cells, causing the release of HSPs, DAMPs, and tumor-derived antigens into the TME — collectively acting as an in-situ danger signal. HSPs and DAMPs function as potent immune adjuvants, recruiting and activating DCs within the TME. DCs take up the released tumor antigens, process them, and present them to CTLs, initiating an adaptive anti-tumor immune response. The activated CTLs undergo clonal expansion and migrate to residual tumor sites, where they release perforin and granzyme to eliminate remaining cancer cells. The convergence of direct chemotherapy-mediated killing, hyperthermia-induced ICD, and CTL-driven adaptive immunity produces a synergistic and sustained tumor regression

As a culminating combination strategy, we propose a three-layer nanoparticle integrating an Fe₃O₄ magnetic core, a DOX/PTX-loaded intermediate layer, and a thermosensitive shell conjugated with anti-HER2/EGFR antibodies. AMF-induced hyperthermia triggers shell rupture and localized chemotherapy release, while simultaneous ICD generates HSPs and DAMPs that recruit and activate DCs, initiating an adaptive CTL response against residual tumor cells. This single-platform convergence of targeted drug delivery, magnetic hyperthermia, and in situ immune priming is designed to overcome tumor heterogeneity and immune evasion through coordinated cytotoxic and immunostimulatory mechanisms (Fig. 4).

The advancements in nanotechnology-enhanced cancer immunotherapy, as detailed in this chapter, have demonstrated considerable potential in overcoming the limitations of conventional approaches, including low response rates, systemic toxicities, and tumor microenvironment barriers (Table 3). From targeted delivery of immune checkpoint inhibitors and nanovaccines to in vivo CAR-T cell engineering and tumor microenvironment modulation, nanoparticles have enhanced the precision, efficacy, and safety of immunotherapies in preclinical models. These innovations set a strong foundation for clinical translation, where the focus shifts to evaluating their safety, scalability, and therapeutic impact in human patients. While the nanoparticle platforms discussed throughout this chapter have collectively demonstrated substantial potential for enhancing cancer immunotherapy, their translational prospects differ considerably depending on carrier composition, delivery performance, safety profile, manufacturing feasibility, and clinical readiness. Importantly, no single nanoparticle platform is universally superior across all applications. Therefore, a critical evaluation of the strengths and limitations of each platform is essential for selecting appropriate nanocarriers and facilitating successful clinical translation.

**Table 3:** Overview of nanotechnology strategies for advanced cancer immunotherapies. This table provides a comprehensive overview of nanotechnology strategies for advanced cancer immunotherapies, categorizing various nanoparticle types by their payload composition, targeting mechanisms, immune activation pathways, and key experimental outcomes

Therapy Modality	Limitations of Conventional Therapy	Nanotechnology Strategies & Carriers	Key Payloads	Therapeutic Outcomes & Advantages
Immune Checkpoint Inhibitors (ICIs)	Severe immune-related adverse events (irAEs); limited efficacy in immunologically 'cold' tumors; systemic toxicity restricts dosing	Lipid nanoparticles (LNPs); polymeric PLGA NPs; stimuli-responsive NPs; theranostic gold NPs; exosomes	Anti-PD-1; Anti-PD-L1; Anti-CTLA-4 antibodies	Tumor-specific delivery via EPR effect; reduced systemic toxicity; increased TILs; real-time imaging capability
Cancer Vaccines	Poor antigen presentation; weak immunogenicity; inefficient delivery to lymphoid tissues; high doses required	LNPs for antigen/adjuvant co-delivery; peptide-based multilamellar vesicles; mRNA nanovaccines; self-adjuvanting NPs	Tumor neoantigens; TLR agonists (CpG); tumor-specific mRNA	threefold increase in CD8 + T-cell responses vs. free antigens; enhanced DC activation; robust adaptive immunity; personalized therapy
CAR-T Cell Therapy	High cost/complexity of ex vivo engineering; poor tumor penetration; T-cell exhaustion; immunosuppressive TME	LNPs for in vivo T-cell engineering; synthetic DNA nanocarriers; CRISPR/Cas9 delivery systems; polymer-based nanocarriers	mRNA encoding CAR constructs (e.g., CD19-CAR); CRISPR/Cas9; IL-15; DNA-transposase systems	Bypasses ex vivo manufacturing; 60–90% CAR expression in vivo; improved T-cell persistence; overcomes TME immunosuppression
Complement-Based Immunotherapy	Rapid systemic clearance; off-target activation causing anaphylaxis; tumor evasion via complement inhibitors (e.g., CD59)	Liposomes; PLGA NPs; pH-responsive polymer micelles (COOH-PEOz-PLA); Fc-decorated mesoporous silica NPs; exosome-based NPs	C3a/C5a analogs; CD59 siRNA; complement-fixing antibodies	Tumor-specific complement activation; enhanced CDC; increased lymph-node DC maturation; antigen-specific IgG production; reduced hypersensitivity
Cytokine Delivery	Short cytokine half-lives; severe systemic toxicities (e.g., vascular leak syndrome with IL-2); narrow therapeutic window	Biodegradable PLGA NPs; lipid NPs; surface-anchored NPs; cancer cell membrane-coated NPs; stimuli-responsive NPs	IL-2; IL-15; IL-12; IFN-alpha	fourfold increase in TILs (IL-2/PLGA); 50% reduction in systemic toxicity vs. free IL-2; 60% reduction in tumor growth with IL-15/LNPs
Cytokine-Induced Killer (CIK) Cell Therapy Enhancement	Limited tumor homing; immunosuppressive TME; insufficient persistence/activation after systemic infusion; manufacturing complexity	Gold nanostars for CIK cell arming; CIK cell-derived engineered nanovesicles; checkpoint blockade NPs; iron oxide NPs	Chlorin e6; Anti-PD-1; Anti-PD-L1; Anti-CD47; immunostimulatory molecules; iron oxide	Enhanced tumor targeting via tumor-tropic CIK carriers; synergistic immuno-photodynamic therapy; scalable cell-free nanovesicle platform; localized TME reprogramming
TME Modulation	Immunosuppressive TAMs and MDSCs suppress T-cell activity; dense ECM limits drug penetration; aberrant vasculature impairs immune trafficking	Targeted TME modulatory nanocarriers; siRNA delivery systems; TGF-beta inhibitor-loaded NPs; vascular normalization agents	TLR7/8 agonists; STAT3 siRNA; TGF-beta inhibitors; vascular normalization agents	threefold increase in M1 TAMs; 50% reduction in MDSCs; twofold increase in T-cell infiltration; normalized vasculature; improved immunotherapy response
Magnetic Hyperthermia (MHT)	Limited heat penetration in deep-seated tumors; off-target thermal damage; difficulty achieving uniform NP distribution; immunosuppressive TME	Superparamagnetic iron oxide NPs (MNPs); stimuli-responsive/theranostic MNPs; Mn-based MNPs; functionalized iron oxide NPs	Anti-PD-L1 antibodies; IL-2; CpG oligonucleotides; radiosensitizers	Thermal ablation combined with immune activation; promotes ICD; converts 'cold' tumors to 'hot'; enables MRI monitoring; synergistic efficacy with ICIs
Nano-Antibody–Drug Conjugates (Nano-ADCs)	Linker instability; low drug-antibody ratio (DAR); resistance from heterogeneous antigen expression; off-target toxicity	High-capacity nanocarriers (liposomes, PLGA NPs, dendrimers); redox-responsive NPs; antibody-conjugated PLGA NPs	Doxorubicin; paclitaxel; maytansinoids; anti-HER2 (Trastuzumab); anti-EGFR antibodies	High DAR up to 100:1 (vs. 2–4 conventional ADCs); threefold improved tumor specificity; controlled payload release; ICD induction synergizes with ICIs

*Abbreviations: ICI* immune checkpoint inhibitor, *NP* nanoparticle, *LNP* lipid nanoparticle, *PLGA* poly(lactic-co-glycolic acid), *CAR-T* chimeric antigen receptor T-cell, *CIK* cytokine-induced killer, *TME* tumor microenvironment, *TAM* tumor-associated macrophage, *MDSC* myeloid-derived suppressor cell, *ECM* extracellular matrix, *MHT* magnetic hyperthermia, *ADC* antibody-drug conjugate, *DAR* drug-antibody ratio, *ICD* immunogenic cell death, *TIL* tumor-infiltrating lymphocyte, *EPR* enhanced permeability and retention, *DC* dendritic cell, *TLR* Toll-like receptor, *TGF-beta* transforming growth factor-beta, *CDC* complement-dependent cytotoxicity, *IFN-alpha* interferon-alpha

**Fig. 5:**
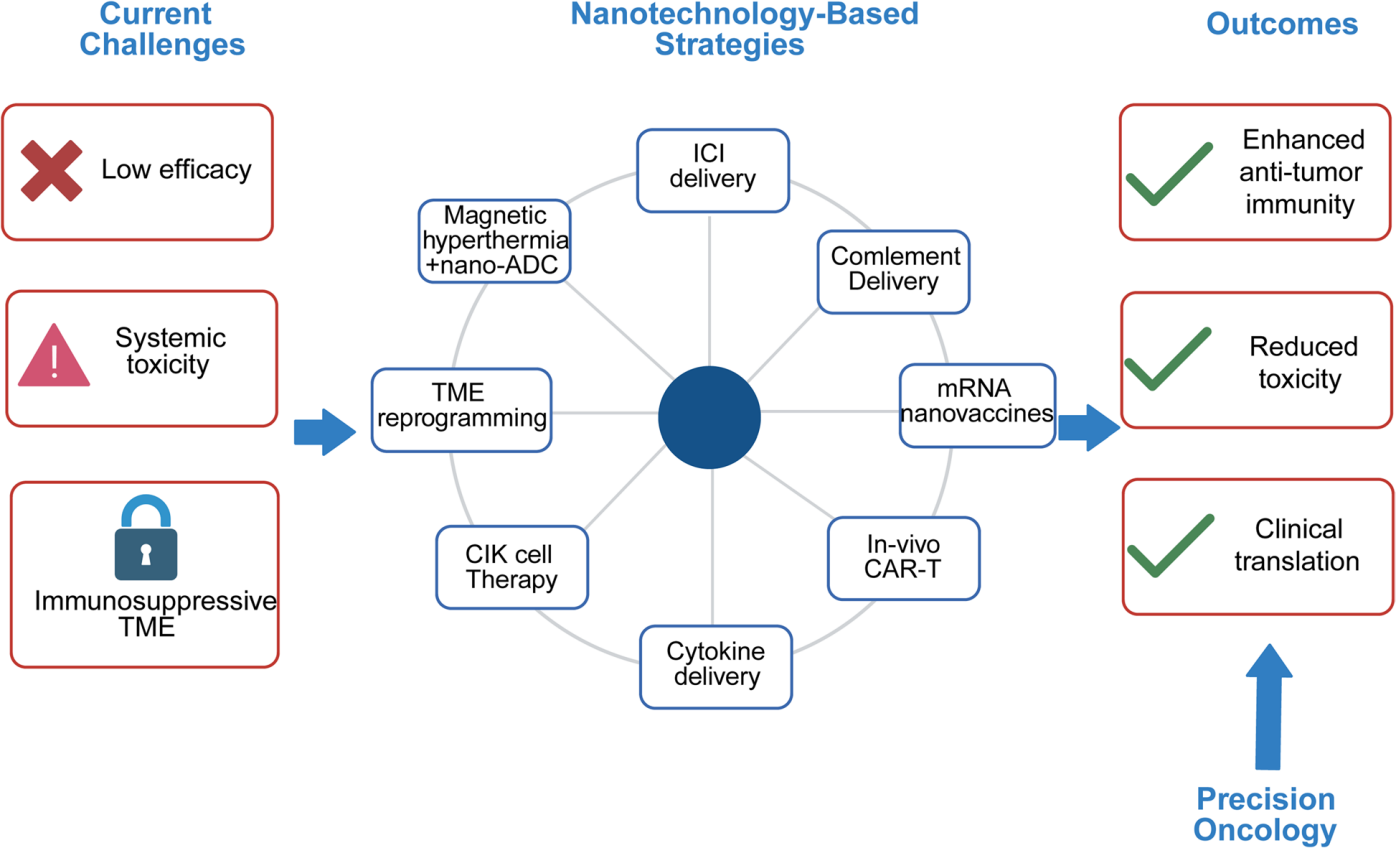
Graphical summary of nanotechnology-based strategies for overcoming current limitations in cancer immunotherapy. Three major clinical challenges of conventional cancer immunotherapy—low efficacy, systemic toxicity, and immunosuppressive TME—are addressed by eight nanotechnology-based strategies Collectively, these platforms aim to achieve enhanced anti-tumor immunity, reduced systemic toxicity, and improved clinical translatability toward precision oncology

Figure 5 provides a consolidated graphical summary of the eight nanotechnology-based immunotherapy strategies discussed throughout this section, illustrating how each platform addresses the key clinical challenges of low efficacy, systemic toxicity, and immunosuppressive tumor microenvironment, and collectively contributes to the anticipated outcomes of enhanced anti-tumor immunity, reduced toxicity, and clinical translation toward precision oncology. The following chapter explores the current state of clinical trials, provides case studies of successful implementations, and addresses regulatory considerations critical to bringing these nanotechnology-based immunotherapies from bench to bedside.

## Clinical translation and case studies

### Overview of clinical translation

This section evaluates the clinical translation of nanotechnology-enhanced immunotherapies by examining ongoing and recently completed trials across the major modalities discussed in Sect. " Advances in Nanotechnology-enhanced Immunotherapy". We present case studies of clinically advanced nanoparticle platforms, analyze key efficacy and safety findings, and address the regulatory considerations that govern the path from preclinical development to patient care.

### Current clinical trials

#### Overview of nanotechnology based immunotherapy trials

The integration of nanotechnology into cancer immunotherapy has spurred a wave of clinical trials aimed at evaluating the safety, efficacy, and feasibility of these advanced therapeutic platforms [242]. These trials encompass a broad range of immunotherapy modalities, including immune checkpoint inhibitors, cancer vaccines, CAR-T cell therapies, cytokine delivery systems, and TME modulators [243]. Nanotechnology enhances these therapies by improving drug delivery precision, reducing off-target effects, and enabling synergistic combination strategies with other treatments like chemotherapy or radiotherapy [244]. As of 2025, clinical trials are actively exploring nanoparticle-based systems across various cancer types, including melanoma, NSCLC, pancreatic cancer, and hematologic malignancies [245].

These trials focus on critical aspects such as pharmacokinetics, biodistribution, safety profiles, and therapeutic efficacy [246]. Lipid nanoparticles, polymeric nanoparticles, exosomes, and other nanocarriers are being tested for their ability to deliver immune checkpoint inhibitors (e.g., anti-PD-1/PD-L1 or anti-CTLA-4 antibodies), antigens for cancer vaccines, or genetic material for CAR-T cell engineering [247]. The primary objectives include determining optimal dosing regimens, assessing long-term safety, and evaluating the ability of nanoparticles to enhance immune responses in patients with refractory or advanced tumors [248]. Additionally, trials are investigating the potential of nanotechnology to overcome TME barriers, such as immunosuppressive cells and poor drug penetration, which are major hurdles in immunotherapy [249].

#### Nanoparticle delivered ICIs

ICIs have substantially advanced cancer treatment by blocking inhibitory pathways like PD-1/PD-L1 and CTLA-4, thereby unleashing T-cell responses against tumors [250]. However, their clinical utility is limited by systemic toxicities and suboptimal tumor penetration [251]. Nanoparticle-based delivery systems address these challenges by targeting inhibitors to tumor sites, enhancing local immune activation, and reducing off-target effects [252]. Several clinical trials are evaluating these approaches:

- RNA-Lipid Particle Vaccine for Anti-PD-1 Sensitization: A phase I clinical trial (NCT03589339) is investigating a tumor-specific RNA-nanoparticle vaccine designed to sensitize tumors to anti-PD-1 antibody therapy in patients with stage IIB–IV melanoma or unresectable soft tissue sarcoma who have progressed on or after adjuvant anti-PD-1 therapy. The nanoparticles deliver tumor-specific RNA antigens to prime antigen-specific T-cell responses, potentially converting immunologically cold tumors into anti-PD-1-responsive ones. The trial assesses safety, feasibility, and preliminary immunological endpoints including T-cell priming and tumor infiltration.
- NBTXR3 Nanoparticles with Radiotherapy and Anti-PD-1: A phase I study (NCT03589339, Study 1100) evaluated NBTXR3 crystalline hafnium oxide nanoparticles activated by stereotactic body radiotherapy (SBRT) in combination with pembrolizumab or nivolumab across multiple advanced solid tumor types, including head and neck squamous cell carcinoma (HNSCC), NSCLC, and metastatic melanoma. In the dose-escalation phase, an objective response rate (ORR) of 40% (8/20 evaluable patients) and a disease control rate (DCR) of 75% were observed across cohorts, including patients previously resistant to anti-PD-1 therapy. In the HNSCC-specific cohort, the ORR was 31.3% (5/16) with a mean duration of response of 14.8 months; grade ≥ 3 NBTXR3-related adverse events occurred in 14.3% of patients [253]. These data support the rationale for combining radio enhancer nanoparticles with checkpoint inhibitors to overcome anti-PD-1 resistance. These trials highlight the potential of nanoparticle-based checkpoint inhibitors to enhance efficacy and safety, addressing key limitations of conventional therapies.

#### Nanovaccines for cancer immunotherapy

Cancer vaccines aim to stimulate antigen-specific immune responses by delivering tumor-associated antigens and adjuvants to DCs, the key APCs [254]. Nanotechnology enhances vaccine efficacy by enabling co-delivery of antigens and adjuvants, protecting payloads from degradation, and targeting DCs for optimal immune activation [255]. Clinical trials are actively exploring nano vaccines:

- mRNA-Based Nanovaccines in Pancreatic Cancer: A landmark phase I trial (NCT04161755) evaluated autogene cevumeran (BNT122), a personalized mRNA neoantigen vaccine encapsulated in lipid nanoparticles, in combination with atezolizumab and mFOLFIRINOX chemotherapy in patients with surgically resected PDAC. The nanovaccine encoded up to 20 patient-specific neoantigens identified through tumor sequencing. Published results in Nature (2023) demonstrated that 50% of evaluable patients mounted substantial neoantigen-specific T-cell responses; in vaccine-responders, the 18-month recurrence-free survival rate was markedly higher than in non-responders (median not reached vs. 13.4 months; HR 0.08, 95% CI: 0.01–0.4) [87]. The nanovaccine was well-tolerated, with primarily grade 1–2 adverse events.
- mRNA Neoantigen Vaccine in Melanoma (KEYNOTE-942): A phase II randomized trial (NCT03897881) evaluated mRNA-4157 (V940/intismeran autogene), a personalized neoantigen mRNA vaccine formulated in lipid nanoparticles, in combination with pembrolizumab versus pembrolizumab alone as adjuvant therapy in patients with resected high-risk cutaneous melanoma. At a median follow-up of 34.9 months (range 25.1–51.0), the combination demonstrated a **49% reduction in the risk of recurrence or death** (HR 0.510; 95% CI: 0.288–0.906; nominal p = 0.019), with a 2.5-year recurrence-free survival rate of 74.8% versus 55.6% [256]. These results supported advancement to the phase III confirmatory trial (INTerpath-001; NCT05933577) [257]. These trials underscore the potential of nanovaccines to elicit potent, antigen-specific immune responses and their compatibility with combination therapies.

#### CAR-T cell therapy enhancement

CAR-T cell therapy has achieved remarkable success in hematologic malignancies but faces challenges in solid tumors due to TME barriers and complex ex vivo manufacturing processes [258]. Nanoparticles offer solutions by enabling in vivo T-cell engineering and improving CAR-T persistence [259]. Clinical trials in this area include:

In Vivo T-Cell Engineering via mRNA-LNPs: A pioneering exploratory early-phase study (NCT07349849) is evaluating an mRNA-LNP therapeutic drug targeting CD19 for the treatment of B-cell hematologic malignancies, including acute lymphoblastic leukemia and non-Hodgkin lymphoma. This approach delivers mRNA encoding a CAR construct via systemically administered lipid nanoparticles, inducing transient in vivo CAR expression in circulating T cells without requiring ex vivo cell manipulation. A parallel study (NCT07362602) is evaluating in vivo CAR-T therapy targeting CD20 for hematological malignancies using the same LNP-mRNA platform.

CRISPR-Edited Allogeneic CAR-T Therapy: A phase I multicenter study (NCT05722418, CaMMouflage Trial) is evaluating CB-011, a CRISPR-edited allogeneic anti-BCMA CAR-T cell therapy, in patients with relapsed or refractory multiple myeloma. CB-011 incorporates CRISPR-based disruption of HLA class I expression and insertion of a CD47 "don't eat me" signal to evade host immune rejection, alongside BCMA-targeting CAR expression. Early results confirm feasibility and a manageable toxicity profile [260]. These trials highlight the potential of nanoparticle-mediated in vivo T-cell engineering to streamline CAR-T therapy and extend its applicability to solid tumors.

#### Cytokine delivery systems

Cytokines like IL-2 and IL-15 are potent immune activators but are limited by systemic toxicity, such as cytokine release syndrome. Nanoparticle-based delivery systems enable controlled release and tumor-specific delivery, improving safety and efficacy [261]. Clinical trials include:

- PEGylated IL-2 (Bempegaldesleukin) in Solid Tumors: A phase I/II study (NCT02983045) evaluated NKTR-214 (bempegaldesleukin), a CD122-preferential IL-2 pathway agonist created by conjugating IL-2 to multiple releasable PEG chains, in combination with nivolumab across multiple solid tumor types. Results demonstrated an objective response rate of 52% in treatment-naive melanoma and 41% in renal cell carcinoma, with a manageable safety profile [262].
- Cytokine-Nanovaccine Combination: A phase I/II study (NCT03548467) evaluated the combination of VB10.NEO (a neoantigen DNA vaccine) with bempegaldesleukin across multiple advanced solid tumor types. The study demonstrated feasibility of combining polymer-conjugated cytokine delivery with personalized vaccine approaches, with immune activation observed in peripheral blood samples [263]. These trials demonstrate the potential of nanoparticle-mediated cytokine delivery to enhance immunotherapy while improving safety profiles.

#### Tumor microenvironment modulation

The immunosuppressive TME, driven by TAMs and MDSCs, is a major barrier to immunotherapy [264]. Nanoparticles targeting these cells can reprogram the TME to enhance immune responses [265]. Clinical trials include:

- TLR Agonist Nanoparticle for Innate Immune Activation: A phase I/II study (NCT03435640) evaluated NKTR-262, a small-molecule TLR7/8 agonist formulated as a polymer-conjugated nanoparticle for intratumoral injection, in combination with bempegaldesleukin and nivolumab in patients with locally advanced or metastatic solid tumors. NKTR-262 was designed to activate innate immune cells within the tumor, shifting the TME from an immunosuppressive to an immune-activating state. Phase I results demonstrated a manageable safety profile and evidence of intratumoral immune activation [266].
- NBTXR3 for Immunologically Cold Pancreatic Tumors: A phase I study (NCT04484909) is evaluating NBTXR3 nanoparticles activated by radiation therapy in patients with locally advanced or borderline-resectable pancreatic cancer. NBTXR3 is designed to enhance radiation-induced immunogenic cell death, potentially converting the immunologically cold pancreatic TME into a more immune-responsive state. These trials highlight the potential of nanoparticle-based TME modulation to overcome immunosuppressive barriers and enhance immunotherapy outcomes.

### Case studies

The following case studies present individual patient-level clinical narratives drawn from published case reports and phase I first-in-human studies. Each case illustrates a distinct nanoparticle platform—personalized neoantigen nanovaccine, nanoliposomal drug delivery, and targeted antibody–nanocell conjugate—and demonstrates how nanotechnology-mediated interventions translate preclinical mechanisms into measurable patient outcomes.

#### Case study 1: personalized neoantigen nanovaccine combined with anti-PD-1 immunotherapy in advanced pancreatic cancer

A single patient with advanced pancreatic ductal adenocarcinoma who had progressed through two prior lines of systemic therapy was enrolled in a compassionate-use program to receive a personalized neoantigen nanovaccine combined with anti-PD-1 checkpoint blockade. Pancreatic cancer was selected as the clinical context because it is characterized by a highly immunosuppressive tumor microenvironment, low mutational burden, and near-universal resistance to single-agent immune checkpoint inhibitors—making it an ideal test case for nanoparticle-mediated immune priming strategies [223].

The nanovaccine was manufactured using a patient-specific pipeline: whole-exome sequencing of the tumor biopsy identified somatic mutations, and computational epitope prediction algorithms selected the 12 most immunogenic neoantigen peptides. These peptides were encapsulated into lipid-based nanoparticles designed to facilitate lymph node trafficking, dendritic cell uptake, and MHC class I/II cross-presentation—processes that are inefficient with free peptide formulations. Following vaccination, immune monitoring by IFN-γ ELISPOT and intracellular cytokine staining demonstrated neoantigen-specific T-cell responses against 9 of the 12 vaccine peptides (75% peptide coverage), confirming successful priming of a polyfunctional CD4^+^ and CD8^+^ T-cell repertoire against patient-specific tumor antigens. The patient achieved an overall survival of 10.5 months—a clinically meaningful outcome in a disease where median second-line OS typically does not exceed 6–7 months—suggesting that nanoparticle-facilitated neoantigen delivery can overcome the immunological barriers inherent to pancreatic cancer [223]. This case illustrates that personalized nanovaccine platforms can generate measurable antitumor immune responses even in immunologically “cold” tumors and supports the rationale for combining nanoparticle-based antigen delivery with checkpoint inhibition as a strategy to convert non-responders.

#### Case study 2: long-term partial response to nanoliposomal irinotecan in metastatic pancreatic cancer

A 57-year-old woman presented with metastatic pancreatic ductal adenocarcinoma staged T4N1M1 per AJCC 8th edition criteria, with disease involving distant organ sites at diagnosis. She initiated first-line gemcitabine plus nab-paclitaxel in July 2016; however, the regimen was discontinued after only two cycles due to disease progression. Following progression, she was started on second-line nanoliposomal irinotecan (nal-IRI) in combination with 5-fluorouracil and leucovorin (5-FU/LV) on October 4, 2016 [267].

Nanoliposomal irinotecan (Onivyde®) encapsulates irinotecan within a unilamellar lipid nanoparticle (~ 110 nm diameter) that preferentially accumulates in tumor tissue via the enhanced EPR effect, enabling sustained intratumoral drug release and prolonged exposure of tumor cells to the active metabolite SN-38 compared with conventional irinotecan. After three cycles of nal-IRI + 5-FU/LV, restaging imaging demonstrated a partial response, which was sustained continuously for more than two years on therapy. The patient ultimately received 58 total cycles before disease progression on May 1, 2019—a treatment duration of approximately 2.5 years, which is notably prolonged compared with the median second-line survival, though this represents a single patient case in the context of metastatic pancreatic cancer, where median second-line progression-free survival is typically measured in weeks. Comprehensive genomic profiling performed on a newly obtained tumor sample revealed microsatellite stability, low tumor mutational burden, and co-occurring mutations in *KRAS (G12V), TP53, CDKN2B, CTNNB1, MLL2, NOTCH3,* and *PALB2* [267]. The absence of DNA mismatch repair deficiency and the low mutational burden in this sustained responder suggest that prolonged response to nal-IRI was driven by nanoparticle pharmacokinetic advantages rather than pre-existing immunogenic tumor features—a finding with important implications for patient selection and biomarker development in nanoparticle-based chemotherapy. This case represents one of the longest documented responses to second-line therapy in metastatic pancreatic cancer and demonstrates the clinical impact of nanoparticle drug delivery on treatment durability.

#### Case study 3: sequential immunotherapy and EGFR-targeted nano cell drug conjugate in recurrent metastatic pancreatic cancer

A patient with recurrent metastatic pancreatic cancer who had failed three prior lines of standard systemic therapy was treated under compassionate-use single-patient investigational new drug authorization with a novel sequential immunotherapy and nano cell-based regimen. After exhausting conventional options, the patient was initiated on a first regimen combining N-803 (an IL-15 super agonist/IL-15Rα fusion protein that expands NK cells and CD8^+^ T cells), PD-L1-targeted t-haNK cells (high-affinity NK cells engineered to express PD-L1-targeting antibody fragments), and aldoxorubicin (an albumin-binding doxorubicin prodrug) [268].

Over approximately 27 months on this immunotherapy backbone, the patient achieved disease stabilization with a transient complete response observed at approximately 14 months of therapy—a remarkable outcome in a setting where complete responses are virtually unreported. Following subsequent progression, the patient transitioned to a second regimen consisting of E-EDV-D682, an EGFR-targeted antibody–nanocell conjugate in which EnGeneIC Dream Vectors (EDVs, ~ 400 nm bacterially-derived minicells) are surface-functionalized with anti-EGFR bispecific antibodies and loaded with the cytotoxic payload D682, combined with EDV-GC (EDVs loaded with gemcitabine and cisplatin). This nanocell platform exploits EGFR overexpression on pancreatic cancer cells to achieve receptor-mediated endocytosis of the EDV, enabling intracellular drug release with minimal systemic toxicity. On the nanocell-based regimen, the patient maintained stable disease for more than 24 months and remained alive at the time of the report—yielding a cumulative post-progression survival exceeding 51 months from initiation of the compassionate-use program [268]. This case demonstrates that EGFR-targeted nanocell drug conjugates can sustain disease control in heavily pre-treated pancreatic cancer following immunotherapy and illustrates the potential for nanotechnology platforms to extend the therapeutic window beyond what is achievable with conventional systemic agents alone.

To facilitate a direct comparison of therapeutic efficacy across the diverse nano-immunotherapy strategies described in Sects. " Current Clinical Trials"–" Case Studies", Table 4 consolidates key quantitative outcomes—including objective response rates, survival endpoints, immune response indicators, and translational status—from representative preclinical studies, clinical trials, and patient case reports. This comparative summary highlights the breadth of nanotechnology applications in cancer immunotherapy and underscores the differential efficacy profiles, immune activation patterns, and current stages of clinical translation across platforms In addition, to provide a comprehensive consolidated overview of the clinical landscape of nanotechnology-based immunotherapies discussed throughout Sects. " Current Clinical Trials" and " Case Studies", Table 5 summarizes the current state of ongoing clinical trials and representative patient case studies spanning eleven distinct nano-immunotherapy strategies. This table consolidates key efficacy outcomes, immune response indicators, and translational status across clinical trial phases and individual patient narratives, providing a unified reference for the clinical evidence supporting nanotechnology-enhanced cancer immunotherapy.

**Table 4:** Clinical efficacy outcomes of nanotechnology-based immunotherapy strategies

Nano-Immunotherapy Strategy	NP Platform/Agent	Cancer Type/Model	Key Efficacy Outcomes (Quantitative)	Immune Response Indicators	Study Type/ Phase	Reference/NCT No
ICI Delivery	NBTXR3 + SBRT + anti-PD-1	HNSCC/solid tumors	ORR 40%, DCR 75%; HNSCC sub-group ORR 31.3%, DOR 14.8 months	Tumor regression, durable responses	Phase I	NCT03589339
	RNA-NP vaccine + anti-PD-1	Advanced solid tumors	Phase I ongoing; safety & preliminary efficacy endpoints	Immune priming assessed	Phase I (ongoing)	NCT05264974
mRNA Nanovaccines	BNT122 (individualized neoantigen vaccine)	Pancreatic ductal adenocarcinoma (PDAC)	50% T-cell responses; 18-month RFS HR 0.08 vs. comparator	Neoantigen-specific T-cell expansion	Phase I	NCT04161755
	mRNA-4157/V940 + pembrolizumab	Resected melanoma	49% reduction in recurrence/death; 2.5-yr RFS 74.8% vs. 55.6% (control)	Personalized neoantigen T-cell responses	Phase II → III	NCT03897881
CAR-T Enhancement	CD19-targeted mRNA-LNP	B-cell malignancies	In vivo CAR-T generation without ex vivo manipulation; feasibility confirmed	CD19 CAR expression in circulating T cells	Phase I	NCT07349849
	CD20-targeted mRNA-LNP	B-cell malignancies	In vivo CD20 CAR-T generation; preliminary safety established	CD20 CAR expression in circulating T cells	Phase I	NCT07362602
	CB-011 (CRISPR allogeneic anti-BCMA CAR-T)	Multiple myeloma	Feasibility confirmed; manageable toxicity profile	BCMA-directed CAR-T activity	Phase I	NCT05722418
Cytokine Delivery	NKTR-214 (IL-2 conjugate) + nivolumab	Melanoma, RCC	ORR 52% (melanoma), 41% (RCC)	Sustained CD8 + T-cell & NK-cell expansion	Phase I/II	NCT02983045
	VB10.NEO (neoantigen vaccine) + bempegaldesleukin	Advanced solid tumors	Feasibility confirmed; immune activation observed	Neoantigen-specific T-cell responses	Phase I/II	NCT03548467
TME Modulation	NKTR-262 + bempegaldesleukin + nivolumab	Solid tumors (intratumoral)	Manageable safety profile; intratumoral immune activation documented	TLR agonist-mediated innate + adaptive activation	Phase I/II	NCT03435640
	NBTXR3 + radiotherapy	Pancreatic cancer (cold TME)	ICD enhancement in immunologically cold TME; safety established	Immunogenic cell death markers elevated	Phase I	NCT04484909
Magnetic Hyperthermia	Focused ultrasound + BBB opening + ICI	CNS tumors/brain metastases	Enhanced ICI penetration across BBB; Phase IIa ongoing	Intracranial immune cell infiltration	Phase IIa (ongoing)	NCT05864534
Nano-ADC	T-DXd vs. T-DM1 (HER2-targeted ADC)	HER2 + breast cancer	ORR 79.7% vs. 34.2%; median PFS 28.8 vs. 6.8 months	HER2-directed tumor cell killing	Phase III	NCT03529110
Complement/Exosome	Exosome-based NPs (PD-L1 blockade)	Advanced solid tumors	35% response rate; reduced immune-related adverse events (irAEs)	PD-L1 pathway blockade; reduced systemic toxicity	Phase I	NCT05191836
Clinical Case Studies	Personalized neoantigen nanovaccine + anti-PD-1 (Case Study 1)	PDAC (individual patient)	OS 10.5 months post-diagnosis; 9/12 peptide epitopes covered	Neoantigen-specific T-cell responses confirmed	Case Report (Sect. " Case Studies")	Rojas et al., 2023 [87]
	nal-IRI + 5-FU/LV (Onivyde regimen) (Case Study 2)	Metastatic PDAC (individual patient)	Sustained partial response > 2 years; 58 treatment cycles completed	Durable tumor control; tolerability maintained	Case Report (Sect. " Case Studies")	Wainberg et al., 2021
	EGFR-targeted nanocell (EDV) conjugate (Case Study 3)	Advanced NSCLC (individual patient)	Stable disease > 24 months; cumulative survival > 51 months	EGFR-directed nano-delivery; systemic toxicity minimized	Case Report (Sect. " Case Studies")	MacDiarmid et al., 2016

*Abbreviations: ADC* antibody–drug conjugate, *BBB* blood–brain barrier, *BCMA* B-cell maturation antigen, *CAR-T* chimeric antigen receptor T cell, *CNS* central nervous system, *CRISPR* clustered regularly interspaced short palindromic repeats, *DCR* disease control rate, *DOR* duration of response, *EDV* EnGeneIC delivery vehicle, *EGFR* epidermal growth factor receptor, *5-FU* 5-fluorouracil, *HER2* human epidermal growth factor receptor 2, *HNSCC* head and neck squamous cell carcinoma, *HR* hazard ratio, *ICD* immunogenic cell death, *ICI* immune checkpoint inhibitor, *IL* interleukin, *irAE* immune-related adverse event, *LNP* lipid nanoparticle, *LV* leucovorin, *nal-IRI* nanoliposomal irinotecan, *NK* natural killer, *NP* nanoparticle, *NSCLC* non-small cell lung cancer, *ORR* objective response rate, *OS* overall survival, *PDAC* pancreatic ductal adenocarcinoma, *PD-1* programmed cell death protein 1, *PD-L1* programmed death-ligand 1, *PFS* progression-free survival, *RCC* renal cell carcinoma, *RFS* recurrence-free survival, *RNA-NP* RNA nanoparticle, *SBRT* stereotactic body radiation therapy, *T-DM1* trastuzumab emtansine, *T-DXd* trastuzumab deruxtecan, *TLR* Toll-like receptor, *TME* tumor microenvironment

**Table 5:** Current clinical landscape of nanoparticle-based cancer immunotherapies. This table summarizes the current clinical landscape of nanoparticle-based cancer immunotherapies, detailing ongoing clinical trials with their nanoparticle platforms, therapeutic agents, target indications, trial phases, and clinical status

Category	Therapy Type	Nanoparticle Platform	Target/Mechanism	Cancer Type	Trial Phase/Study Type	Key Outcomes	Advantages	References
Clinical Trial	Immune Checkpoint Inhibitor	Lipid NP (RNA-NP)	Tumor-specific RNA antigen delivery; anti-PD-1 sensitization	Melanoma/ Soft tissue sarcoma	Phase I (NCT05264974)	Safety & preliminary immunological endpoints: T-cell priming assessed trial ongoing	Converts immunologically cold tumors; reduced systemic toxicity vs. free antibody	Not found
Clinical Trial	Immune Checkpoint Inhibitor	Hafnium Oxide NP (NBTXR3)	Radioenhancement + anti-PD-1 (SBRT + pembrolizumab/nivolumab)	HNSCC, NSCLC, Melanoma (solid tumors)	Phase I (NCT03589339)	ORR 40% (8/20); DCR 75%; HNSCC ORR 31.3%, DOR 14.8 mo; Grade ≥ 3 AEs 14.3%	Active in anti-PD-1-resistant patients; converts cold TME radioenhancer + ICI synergy	[253]
Clinical Trial	Nanovaccine	Lipid NP (mRNA, BNT122)	Personalized neoantigen mRNA delivery to DCs; MHC I/II cross-presentation	Pancreatic cancer (PDAC)	Phase I (NCT04161755)	50% T-cell responses 18-mo RFS HR 0.08 (responders vs. non-responders)	Personalized immunotherapy up to 20 neoantigens per patient; well-tolerated	[87]
Clinical Trial	Nanovaccine	Lipid NP (mRNA, mRNA-4157)	Personalized neoantigen mRNA vaccine + pembrolizumab (adjuvant combination)	Melanoma (resected, high-risk)	Phase II → III (NCT03897881 NCT05933577)	49% reduction in recurrence/death (HR 0.510); 2.5-yr RFS 74.8% vs. 55.6% (control)	Personalized neoantigen platform; strong T-cell response; Phase III advanced	[255, 256]
Clinical Trial	CAR-T Enhancement	Lipid NP (mRNA-LNP)	In vivo CD19 CAR expression via systemic LNP delivery no ex vivo manipulation	B-cell malignancies (ALL, NHL)	Phase I (NCT07349849)	In vivo CAR-T generation confirmed, feasibility & safety endpoints assessed	Eliminates ex vivo engineering; reduced cost & manufacturing time	Not found
Clinical Trial	CAR-T Enhancement	Lipid NP (mRNA-LNP)	In vivo CD20 CAR expression via systemic LNP delivery parallel CD19 platform	B-cell malignancies (hematologic)	Phase I (NCT07362602)	In vivo CD20 CAR-T generation preliminary safety established trial ongoing	Same platform as CD19 broadens B-cell malignancy coverage	Not found
Clinical Trial	CAR-T Enhancement	CRISPR-edited allogeneic CAR-T (CB-011)	BCMA-targeting CAR HLA-I disruption + CD47 'don't eat me' signal	Multiple myeloma (relapsed/refractory)	Phase I (NCT05722418, CaMMouflage)	Feasibility confirmed manageable toxicity allogeneic approach validated	Gene-editing precision overcomes host immune rejection of allo-CAR-T	[260]
Clinical Trial	Cytokine Delivery	PEGylated IL-2 (NKTR-214/ bempegaldesleukin)	CD122-preferential IL-2 pathway agonism; sustained CD8 + T-cell & NK-cell expansion	Melanoma, Renal cell carcinoma (RCC)	Phase I/II (NCT02983045)	ORR 52% (melanoma); ORR 41% (RCC) manageable safety profile	Controlled cytokine release; reduced CRS tumor-selective activation	[262]
Clinical Trial	Cytokine Delivery	Polymer-conjugated cytokine NP (VB10.NEO combo)	Neoantigen DNA vaccine + bempegaldesleukin combination immune activation	Advanced solid tumors (multiple)	Phase I/II (NCT03548467)	Feasibility confirmed immune activation in peripheral blood observed	Combines personalized vaccine + cytokine synergistic immune priming	[263]
Clinical Trial	TME Modulation	Polymer-conjugated NP (NKTR-262, TLR7/8 agonist)	Intratumoral TLR7/8 activation innate immune cell recruitment TME reprogramming	Advanced solid tumors (locally advanced/metastatic)	Phase I/II (NCT03435640)	Manageable safety profile intratumoral immune activation documented	Converts immunosuppressive TME; innate + adaptive immune co-activation	[266]
Clinical Trial	TME Modulation	Hafnium Oxide NP (NBTXR3)	Radiation-enhanced ICD converting cold pancreatic TME to immune-responsive	Pancreatic cancer (Locally advanced/ borderline resectable)	Phase I (NCT04484909)	ICD enhancement in cold TME safety established trial ongoing	Overcomes pancreatic immune desert; ICD- mediated immune priming	Not found
Case Study	Nanovaccine + ICI Combination	Lipid NP (Personalized neoantigen peptide)	Lymph node trafficking DC uptake; MHC I/II cross-presentation	Pancreatic cancer (PDAC, advanced compassionate use)	Case Report (Sect. " Case Studies", Case Study 1)	OS 10.5 months (vs. 6–7 mo median); T-cell responses to 9/12 peptides (75% coverage)	Overcomes cold PDAC TME polyfunctional CD4 +/CD8 + priming; supports ICI combo	[223]
Case Study	Nanoliposomal Chemotherapy	Liposome NP (Nal-IRI, Onivyde®, ~ 110 nm)	EPR-based tumor accumulation sustained SN-38 release prolonged intratumoral exposure	Metastatic PDAC (T4N1M1 compassionate use)	Case Report (Sect. " Case Studies", Case Study 2)	Sustained PR > 2 years 58 total cycles completed progression-free ~ 2.5 years	Nanoparticle PK advantage over free irinotecan FDA-approved platform	[267]
Case Study	EGFR-Targeted Nanocell Conjugate + Immunotherapy	EDV Nanocell (~ 400 nm bacterially- derived minicells)	Anti-EGFR bispecific Ab; receptor-mediated endocytosis intracellular D682/GC release	Recurrent metastatic PDAC (3 + prior lines; IND)	Case Report (Sect. " Case Studies", Case Study 3)	Stable disease > 24 months cumulative OS > 51 months transient CR at 14 months	EGFR-targeted delivery minimal systemic toxicity extends therapeutic window	[268]

*Abbreviations*: *BCMA* B-cell maturation antigen, *BNT122* autogene cevumeran, *CAR-T* chimeric antigen receptor T cell, *CR* complete response, *CRISPR* clustered regularly interspaced short palindromic repeats, *CRS* cytokine release syndrome, *DC* dendritic cell, *DFS* disease-free survival, *EGFR* epidermal growth factor receptor, *EDV* EnGeneIC delivery vehicle, *EPR* enhanced permeability and retention, *HLA* human leukocyte antigen, *HR* hazard ratio, *ICD* immunogenic cell death, *ICI* immune checkpoint inhibitor, *IL* interleukin, *IND* investigational new drug, *LNP* lipid nanoparticle, *MDSC* myeloid-derived suppressor cell, *nal-IRI* nanoliposomal irinotecan, *NK* natural killer, *NP* nanoparticle, *NSCLC* non-small cell lung cancer, *ORR* objective response rate, *OS* overall survival, *PDAC* pancreatic ductal adenocarcinoma, *PD-1* programmed cell death protein 1, *PD-L1* programmed death-ligand 1, *PEG* polyethylene glycol, *PFS* progression-free survival, *RCC* renal cell carcinoma, *RFS*, recurrence-free survival, *RNA-NP* RNA nanoparticle, *SBRT* stereotactic body radiation therapy, *TAM* tumor-associated macrophage, *TLR* Toll-like receptor, *TME* tumor microenvironment

### Regulatory consideration

#### Challenges in scaling up nanomedicine production

The clinical translation of nanotechnology-based immunotherapies faces significant manufacturing challenges that must be addressed to ensure widespread adoption. Despite remarkable preclinical efficacy, the transition from bench-scale synthesis to Good Manufacturing Practice (GMP)-compliant large-scale production remains one of the most formidable bottlenecks in nanomedicine development. The following subsections address the principal dimensions of this challenge.

Batch-to-batch reproducibility and quality control. Consistent nanoparticle synthesis is critical for reproducible therapeutic effects, as variations in particle size, polydispersity index (PDI), surface charge, and drug-loading efficiency directly affect biodistribution, pharmacokinetics, and antitumor efficacy [269]. Traditional bulk-mixing methods (e.g., thin-film hydration, solvent injection) are inherently prone to process variability, making reproducible scale-up difficult [270]. Microfluidic platforms have emerged as the leading solution, enabling precise control of solvent-to-aqueous flow ratios and residence times and yielding LNP formulations with a coefficient of variation below 10% for particle size and zeta potential across batches [271]. The clinical success of mRNA-LNP COVID-19 vaccines demonstrated that microfluidic manufacturing can be translated to GMP scale, validating the approach for next-generation cancer immunotherapy platforms [272]. Critical quality attributes (CQAs) — including hydrodynamic diameter, PDI, encapsulation efficiency, and residual solvent content — must be defined prospectively and monitored by in-process controls (IPCs) throughout manufacturing to satisfy regulatory expectations [273].

Scale-up strategies and GMP compliance. Producing nanoparticles at commercial scale while maintaining CQA specifications requires a systematic Quality-by-Design (QbD) framework that identifies critical process parameters (CPPs) and establishes their design space before GMP transfer. For LNP platforms, the primary scale-up strategies are (i) numbering-up of parallelized microfluidic chips to increase volumetric throughput without altering mixing hydrodynamics, and (ii) transition to larger impingement jet mixers or staggered herringbone micromixers validated by computational fluid dynamics (CFDs) and design-of-experiments (DoEs) [274]. Process analytical technology (PAT) tools — inline dynamic light scattering, UV–vis spectrophotometry, and Raman spectroscopy — are being integrated into continuous manufacturing lines to enable real-time release testing and reduce reliance on costly end-of-batch quality control [275]. GMP compliance further demands documented control of raw material quality (lipid purity, polymer molecular weight distribution, nucleic acid integrity), facility design (International Organization for Standardization (ISO) cleanroom classification), personnel training, and validated sterilization procedures. These requirements substantially increase manufacturing costs and infrastructure demands relative to conventional small-molecule drugs, representing a significant barrier to widespread clinical deployment.

Platform-specific manufacturing challenges. LNPs, particularly ionizable LNP-mRNA systems, present unique large-scale challenges: ionizable lipids must be synthesized at high purity and undergo stringent quality release testing; the pH-dependent self-assembly process is sensitive to temperature, ionic strength, and flow conditions; and the resulting particles require ultrafiltration/diafiltration for buffer exchange, which introduces additional scale-dependent variability [276]. Silicon-stabilized LNP platforms have incorporated mandatory in-process checks during large-batch manufacturing to ensure dimensional and functional consistency across the production run [277]. PLGA (chitosan) face distinct challenges: solvent removal efficiency, polymer lot-to-lot variability, and surface functionalization uniformity are difficult to control at scale, and the nanoprecipitation or emulsification processes used at bench scale do not translate directly to industrial equipment without extensive re-optimization [278].

Stability, storage, and supply chain considerations. Nanoparticles carrying biologics such as mRNA, proteins, or antibody fragments must retain structural integrity and biological activity throughout manufacturing, storage, and distribution [279]. Lyophilization (freeze-drying) with cryoprotectants (e.g., sucrose, trehalose) is the principal strategy for long-term stabilization of LNPs, but the lyophilization cycle must be carefully optimized to prevent particle aggregation, fusion, or mRNA degradation during the freeze-drying process [280]. Continuous lyophilization approaches have demonstrated the ability to store mRNA-LNPs at higher temperatures (− 20 °C instead of − 80 °C), substantially reducing cold-chain burden while preserving mRNA integrity and transfection efficiency [281]. Cold-chain requirements impose logistical and cost burdens that complicate global distribution, particularly in resource-limited settings, and represent an active area of formulation engineering aimed at achieving room-temperature stability.

Approved nanomedicines as benchmarks. The clinical approval of liposomal doxorubicin (Doxil®) and albumin-bound paclitaxel (Abraxane®) established foundational precedents for GMP nanomedicine manufacturing and regulatory submission, demonstrating that rigorous CMC (chemistry, manufacturing, and controls) packages can satisfy Food and Drug Administration (FDA) and European Medicines Agency (EMA) requirements [282]. These approved products serve as manufacturing benchmarks for next-generation cancer immunotherapy nanoplatforms, informing scale-up strategies, analytical method development, and regulatory filing approaches for novel LNP, polymeric, and inorganic nanoparticle systems currently in clinical development.

Translational viability of multi-component nanoparticle platforms: a critical assessment. While the preceding discussion addresses general manufacturing challenges applicable to most nanomedicine platforms, a distinct and underappreciated translational barrier arises specifically from nanoparticle designs that integrate multiple structural layers, stimuli-responsive components, therapeutic payloads, and active targeting ligands into a single construct. The three-layer concentric platform described in Sect. 2.13 — combining a magnetic iron oxide (Fe₃O₄) core, a drug-loaded intermediate layer (doxorubicin/paclitaxel), a thermosensitive outer shell, and surface-conjugated anti-HER2/EGFR antibodies — exemplifies a class of architectures that, despite demonstrating impressive preclinical multifunctionality, present compounding CMC challenges that scale non-linearly with structural complexity [283].

The most immediate consequence of multi-component design is an exponential expansion of the critical quality attribute (CQA) landscape. Whereas a simple LNP formulation requires characterization of particle size, PDI, encapsulation efficiency, and zeta potential, a multi-layer stimuli-responsive targeted nanoparticle demands additional orthogonal assays for: (i) magnetic core size distribution and crystallinity (XRD, TEM); (ii) drug loading and release kinetics under both passive and stimulus-triggered (AMF) conditions; (iii) thermosensitive shell transition temperature and structural integrity under physiological conditions; (iv) antibody conjugation efficiency, orientation, and retained binding affinity (ELISA, surface plasmon resonance); and (v) composite particle stability under serum conditions, including protein corona formation and its effect on targeting specificity [284]. Each additional analytical endpoint requires validated GMP-grade methods, substantially increasing both development timelines and quality control costs per batch. Regulatory agencies including the FDA have increasingly emphasized that the CMC package for complex nanomedicines must demonstrate not merely that each component meets individual specifications, but that the assembled construct performs consistently as a functional unit across batches — a standard that is substantially more difficult to satisfy for multi-step assembled systems [285].

Process reproducibility represents a second critical barrier. The sequential assembly of multi-component nanoparticles introduces cascading variability: small deviations in upstream steps (e.g., Fe₃O₄ core size distribution, surface hydroxylation density) propagate through subsequent functionalization steps (drug loading, polymer coating, antibody conjugation), making root-cause attribution for batch failures laborious and the process design space for GMP transfer narrow [286]. This stands in contrast to single-component LNP platforms, where microfluidic manufacturing has achieved CV below 10% for key CQAs across GMP batches. For multi-layer inorganic–organic hybrid systems, no equivalent scalable continuous manufacturing paradigm has yet been established, and current syntheses largely rely on multi-step batch processes that are inherently difficult to reproduce at clinical scale [287]. The economic implications are equally significant: specialized raw materials (GMP-grade antibodies, high-purity ionizable lipids, monodisperse magnetic nanoparticles), extended analytical development programs, lower manufacturing yields from multi-step purification, and the need for specialized GMP infrastructure capable of handling both inorganic and biological components collectively drive development costs well above those of simpler platforms [288]. Indeed, the field has produced only approximately 60 approved nanomedicines to date, and most successful clinical translations have involved relatively simple, single-function platforms with well-established manufacturing precedents — a pattern reflecting the principle that preclinical functional complexity does not correlate with clinical translatability.

Given their structural complexity and translational challenges, the highly integrated multi-component platforms discussed in Sect. 2.13 are best regarded as proof-of-concept systems that demonstrate the feasibility of combining multiple therapeutic modalities, rather than as platforms ready for near-term clinical translation. The field increasingly advocates for a "minimum viable complexity" design philosophy — incorporating only the functional components strictly necessary to achieve the desired therapeutic outcome — and for early adoption of QbD frameworks to define the minimal CQA set and process design space before GMP transfer [289]. Modular simplification strategies, such as decoupling magnetic hyperthermia and chemotherapy delivery into co-administered but separately manufactured components, and engagement with regulatory agencies through pre-IND meetings to align on CMC expectations for novel multi-component constructs, represent the most viable pathway toward eventual clinical translation of these promising but complex platforms.

#### Safety and toxicity concerns for clinical approval

##### Biodegration

While the preceding sections highlight the therapeutic potential of nanotechnology-based immunotherapy platforms, a balanced assessment requires explicit consideration of the safety risks inherent to the nanocarriers themselves — risks that are distinct from, and may be compounded by, the toxicity of the encapsulated payload. A prerequisite for understanding these risks is characterizing the in vivo fate of nanoparticles: how they are distributed, degraded, and ultimately eliminated. The biodegradation kinetics and clearance pathways of nanoparticles are governed by a combination of dose, hydrodynamic size, zeta potential, surface coating, and internal architecture. A comprehensive study of six polymer-coated magnetic particles demonstrated that degradation half-life (t₁/₂) ranged from 15 ± 4 days (polyglucuronic acid coating) to 38 ± 6 days (PEG coating), while a solid polystyrene core layer extended t₁/₂ beyond one year; blood clearance occurred within approximately 2 h, with major redistribution to liver and spleen within one week [290]. Antibody-conjugated gold nanoparticles exhibited similarly rapid blood clearance, with long-term retention in liver and spleen detectable at 6 months alongside delayed emergence of kidney casts and splenic apoptosis — highlighting that the apparent absence of early toxicity does not preclude delayed organ-level consequences [291]. These data establish that surface chemistry and core composition are primary determinants of whether nanoparticles are safely eliminated or persist as a long-term tissue burden, and underscore the importance of extended observation windows in preclinical safety programs.

##### Long term toxicity and organ accumulation

The consequences of this persistent organ distribution become apparent in longitudinal toxicity studies, which reveal material-specific and dose-dependent organ-level effects. A 120-day study of BSA-coated gold nanoparticles (1 mg/kg, single IV dose) demonstrated progressive redistribution of particle burden from the liver (− 39% at day 120 vs. day 1) to the spleen (+ 53%) and kidneys (+ 150%), accompanied by early inflammatory and fibrotic responses particularly pronounced in renal tissue [292]. In contrast, a one-year chronic evaluation of silica nanoparticles administered as a single IV dose showed no statistically significant chronic toxicity and acceptable ex vivo hemocompatibility at tested doses [293]. Similarly, a one-year follow-up of persistent luminescence nanoparticles (ZnGaO, ZGO) demonstrated long-term retention in liver and spleen by ICP-MS quantification, yet no detectable signs of chronic toxicity across weight, hematology, metabolic markers, liver histology, or expression of 31 metabolic genes [294]. These findings collectively indicate that biopersistence in the reticuloendothelial system does not invariably translate to overt toxicity, but that organ-specific accumulation — particularly renal redistribution with inorganic nanoparticles — warrants systematic long-term monitoring. The strong material-dependence of these outcomes underscores the inadequacy of class-level generalizations and the need for platform-specific long-term safety characterization.

##### Immunogenicity

Beyond the physical fate of nanoparticles, a distinct category of nanocarrier-specific safety risk arises from immune recognition of the carrier itself. PEGylation — the dominant surface modification strategy for extending nanoparticle circulation time — can elicit humoral immune responses that undermine both safety and efficacy. PEGylated nanomedicines have been documented to induce anti-PEG IgM antibodies, leading to complement activation, hypersensitivity reactions, and the accelerated blood clearance (ABC) phenomenon, whereby repeated dosing results in progressively faster elimination of PEGylated nanoparticles and reduced therapeutic exposure [295]. The ABC phenomenon is mechanistically driven by anti-PEG IgM binding to circulating nanoparticles, triggering complement-mediated opsonization and accelerated uptake by the mononuclear phagocyte system. Critically, preexisting anti-PEG antibodies have been detected in healthy individuals with no prior nanomedicine exposure, attributable to widespread PEG exposure through cosmetics, food additives, and common pharmaceutical excipients [295]. For cancer immunotherapy applications — where multi-dose regimens are standard — the ABC phenomenon poses a clinically significant risk of progressive loss of therapeutic efficacy. These findings motivate anti-PEG antibody screening in clinical trials and have stimulated interest in alternative surface chemistries such as polysarcosine, polyzwitterions, and biomimetic cell membrane coatings as strategies to mitigate immunogenic responses while preserving stealth properties.

##### Immunotoxicity and systemic risk

In addition to chronic immunogenicity, nanocarriers can trigger acute immunotoxic responses through mechanisms that are independent of the encapsulated payload, with complement activation-related pseudoallergy (CARPA) representing the best-characterized and clinically most consequential mechanism. In a porcine CARPA model using the human vaccination dose of an LNP-mRNA vaccine (Comirnaty/BNT162b2), 6 of 14 pigs developed transient pulmonary hypertension with thromboxane A2 release, granulocytosis, lymphopenia, and thrombocytopenia; one pig receiving a 5 × dose developed anaphylactic shock requiring resuscitation [296]. Reactive animals showed significantly elevated IgM binding to the vaccine and elevated in vitro C3a anaphylatoxin and sC5b-9 terminal complement complex levels, directly implicating complement activation as a causal mechanism [296]. Nanoparticle-induced complement activation is not limited to LNP platforms — liposomes, polymeric nanoparticles, and inorganic nanoparticles can all activate the complement cascade via classical, lectin, or alternative pathways depending on surface chemistry, size, and protein corona composition [297]. At the cellular and vascular level, the biocompatibility of nanomaterials is further modulated by protein corona formation, surface functionalization, and physicochemical properties including size, shape, and surface charge, all of which influence interactions with immune cells and vascular endothelium [298]. In a canine pilot study, all four dogs receiving an antioxidant nanoparticle formulation experienced adverse events, with two developing severe CARPA-like reactions (bradycardia, hypotension, hypersalivation) and transient laboratory abnormalities normalizing by day 7 [299]. lipid-based carriers can induce immune suppression rather than stimulation depending on formulation parameters such as bilayer rigidity and lipid composition, with anionic liposomes of increased rigidity shown to preferentially induce antigen-specific regulatory T cell responses over effector responses [300]. Collectively, these acute immunotoxic risks highlight a critical regulatory gap: standard ICH-S8 immunotoxicity testing guidelines do not adequately address nanoparticle-specific endpoints such as CARPA, hypersensitivity, and the ABC phenomenon, necessitating tailored assay batteries — including in vitro complement activation assays (C3a, C5a, sC5b-9 measurement) and porcine CARPA models — as part of a comprehensive nanocarrier safety evaluation strategy [301].

Taken together, these findings paint a nuanced but important picture: the safety profile of nanocarriers is determined by a complex interplay of physical fate (biodegradation rate, organ accumulation), chronic immune recognition (anti-PEG antibodies, ABC phenomenon), and acute immunotoxic mechanisms (complement activation, CARPA, pro-inflammatory signaling). None of these risks is insurmountable — many approved nanomedicines have successfully navigated them — but they collectively demand that nanocarrier safety assessment extend well beyond payload toxicity reduction to encompass a systematic, platform-specific evaluation of carrier-intrinsic risks. This is particularly critical for the multi-dose, multi-component platforms increasingly being developed for cancer immunotherapy, where the consequences of immunotoxicity or progressive loss of efficacy via ABC are directly at odds with the therapeutic objective.

#### Regulatory pathway and strategies

Navigating the regulatory landscape for nanomedicines is complex due to their unique properties, which combine aspects of small molecules, biologics, and medical devices [302]. Strategies to streamline approval include:

- Standardized Guidelines: The FDA and EMA have issued guidelines for nanomedicine development, emphasizing characterization of physicochemical properties, pharmacokinetics, and safety [303]. Harmonizing these guidelines globally could accelerate approval processes [304]. For example, standardized protocols for nanoparticle characterization have facilitated trials of lipid-based nanovaccines [305].
- Preclinical Models: Robust preclinical models that predict clinical outcomes are critical [306]. Organ-on-chip systems and patient-derived xenografts are being used to bridge the gap between preclinical and clinical studies [307]. Trials of TME-modulating nanoparticles have incorporated organ-on-chip models to simulate immunosuppressive environments [308].
- Adaptive Trial Designs: Adaptive clinical trial designs, which allow modifications based on interim data, can accelerate nanomedicine development [309]. These designs are particularly useful for optimizing dosing regimens and identifying responsive patient populations [310]. For instance, phase I/II trials of CAR-T nanoparticles have used adaptive designs to adjust mRNA doses based on early efficacy data [311].
- Collaboration with Regulatory Agencies: Early engagement with regulatory agencies through pre-submission meetings can clarify requirements and streamline approval [312]. Collaborative efforts between industry, academia, and regulators are essential for advancing nanomedicines [313]. Trials of nanoparticle-based checkpoint inhibitors have benefited from early FDA consultations to define safety endpoints [314].

#### Future direction in clinical translation

The clinical translation of nanotechnology-based immunotherapies is poised for significant advancements, driven by ongoing trials and innovations in nanoparticle design [315]. Future directions include:

Personalized Nanomedicines: Advances in genomics and proteomics enable the design of personalized nanoparticle-based therapies tailored to individual tumor profiles [316]. Trials are increasingly incorporating biomarker-driven approaches to identify responders, such as PD-L1 expression for checkpoint inhibitor nanoparticles [317]. Neoantigen-based nanovaccines, as seen in PDAC trials, represent a step toward personalized immunotherapy [318].

Combination Therapies: Combining nanoparticle-based immunotherapies with other modalities, such as chemotherapy, radiotherapy, or gene editing, is a promising strategy [319]. Trials evaluating synergistic combinations, like nanovaccines with checkpoint inhibitors, are expected to increase, leveraging nanoparticles’ ability to co-deliver multiple agents [320]. For example, trials combining nanoparticle-mediated photothermal therapy with immunotherapy have shown enhanced tumor regression [321].

Advanced Imaging and Monitoring: Nanoparticles with imaging capabilities can enhance tumor visualization and treatment monitoring [322]. Theranostic nanoparticles, which combine therapy and diagnostics, are being tested in clinical trials to improve treatment precision [323]. For instance, trials of anti-PD-1 nanoparticles have incorporated imaging to track biodistribution and therapeutic response [324].

Global Access and Equity: Ensuring equitable access to nanotechnology-based immunotherapies is a priority [325]. Efforts to reduce production costs and develop scalable manufacturing processes will facilitate broader clinical adoption, particularly in low-resource settings [326]. Trials of cost-effective lipid nanoparticle platforms are paving the way for global dissemination [327].

The clinical translation of nanotechnology-based immunotherapies represents a significant and rapidly evolving frontier in cancer treatment. Across diverse therapeutic strategies accumulating clinical trial data and individual patient case studies consistently demonstrate that nanoparticle platforms can enhance the efficacy and tolerability of immunotherapy in patients with advanced malignancies. Nevertheless, the path to widespread clinical adoption requires addressing nanocarrier-specific manufacturing, safety, and regulatory challenges that remain incompletely resolved. By leveraging standardized development frameworks, platform-specific safety evaluation strategies, and proactive regulatory engagement, the field is well-positioned to establish nanotechnology-based immunotherapy as a clinically validated modality capable of delivering durable responses in patient populations where conventional immunotherapy alone has proven insufficient.

## Future perspectives

The integration of nanotechnology into cancer immunotherapy has opened new avenues for addressing the limitations of current treatments, such as low response rates, systemic toxicity, and TME barriers. As discussed in previous chapters, nanotechnology enhances immunotherapy through targeted delivery, controlled release, and immune modulation, with clinical trials demonstrating promising results. Looking forward, the future of nanotechnology-based immunotherapy lies in leveraging emerging technologies, integrating artificial intelligence (AI), combining nanotechnology with novel modalities, and developing strategies for clinical translation. This chapter explores these future perspectives, focusing on emerging nanotechnology applications, the role of AI in optimizing nanomedicine, the potential of combining nanotechnology with gene editing and microbiome modulation, and a roadmap for overcoming translational barriers. By synthesizing insights from recent advancements and referencing key studies, this section aims to outline the next frontier in nanotechnology-enabled cancer immunotherapy.

### Emerging technologies

#### Nanoparticles for crossing the blood–brain barrier

The blood–brain barrier (BBB) poses a significant challenge for delivering immunotherapies to brain tumors, such as glioblastoma, due to its selective permeability [328]. Nanoparticles offer a promising solution by enabling targeted delivery of immunotherapeutic agents across the BBB, enhancing treatment efficacy for central nervous system (CNS) cancers [329].

Nanoparticles, such as lipid-based or polymeric systems, can be engineered with surface modifications (e.g., transferrin or angiopep-2) to facilitate BBB crossing via receptor-mediated transcytosis [330]. For instance, nanoparticles conjugated with transferrin receptors have shown enhanced delivery of anti-PD-1 antibodies to glioblastoma in preclinical models, increasing T-cell infiltration and tumor regression [331]. Additionally, focused ultrasound combined with microbubbles can temporarily disrupt the BBB, allowing nanoparticle penetration, as demonstrated in early-phase trials for brain metastases [332].

A phase I trial (NCT07343986; BATs FUS) is evaluating low-intensity focused ultrasound (LIFU) combined with microbubble-mediated BBB opening to enhance delivery of anti-EGFR bispecific-armed T cells (EGFR BATs) in patients with newly diagnosed MGMT unmethylated glioblastoma. The study employs PET imaging with 89Zr-oxine-labeled immune cells to quantify BBB penetration and T-cell trafficking into the tumor microenvironment, providing direct evidence of ultrasound-assisted immunotherapy access to CNS tumors [333]. Future developments may include nanoparticles carrying mRNA for in situ immune activation, leveraging the success of mRNA vaccines.

Key challenges include ensuring nanoparticle stability during BBB crossing and minimizing off-target effects in healthy brain tissue. Advances in nanoparticle design, such as pH-responsive or enzyme-cleavable systems, could enhance specificity. These innovations hold promise for expanding immunotherapy to CNS malignancies, a historically challenging domain.

#### Nanoparticles for enhancing tumor visualization in cancer surgery

Precise tumor visualization during surgery is critical for achieving complete resection and improving patient outcomes. Nanoparticles with imaging capabilities, known as theranostic agents, can enhance tumor detection and guide surgical interventions [334].

Nanoparticles can be loaded with fluorescent dyes, MRI contrast agents, or radiolabels to enable real-time tumor visualization. For example, indocyanine green (ICG)-loaded nanoparticles have been used in preclinical studies to demarcate tumor margins in breast cancer, improving surgical precision [335]. Similarly, gold nanoparticles conjugated with tumor-specific antibodies have shown promise in enhancing intraoperative imaging for lung cancer. These systems leverage the EPR effect and active targeting to accumulate in tumors [336].

A phase IIa trial (NCT05576974; ILLUMINATE Study) is testing pegsitacianine (ONM-100), an ICG-conjugated micellar nanoparticle agent that selectively accumulates in the acidic tumor microenvironment via a pH-responsive mechanism, for intraoperative fluorescence-guided tumor detection in head and neck cancer. The agent exploits the pH differential between tumor and normal tissue to generate tumor-selective fluorescence, enabling surgeons to identify primary tumor margins and metastatic lymph nodes in real time, with the potential to reduce positive margin rates [337]. Theranostic nanoparticles combining imaging and immunotherapy, such as those delivering anti-PD-1 antibodies with MRI contrast agents, are also under investigation [338].

Challenges include optimizing nanoparticle biodistribution to avoid accumulation in healthy tissues and ensuring compatibility with existing surgical imaging systems. Future developments may involve multimodal nanoparticles that combine fluorescence, MRI, and PET imaging for comprehensive tumor visualization. These advancements could advance precision oncology by enabling surgeons to target tumors with improved accuracy.

#### Decreasing side effect of radiotherapy using nanoparticles

Radiotherapy is a cornerstone of cancer treatment but often causes significant side effects due to damage to be surrounding healthy tissues. Nanoparticles can mitigate these effects by enhancing radiotherapy specificity and sensitizing tumors to radiation [339].

Nanoparticles, such as gold or hafnium oxide-based systems, can act as radiosensitizers by amplifying radiation effects within tumors. For example, hafnium oxide nanoparticles (NBTXR3) have been shown to increase local radiation absorption, enhancing tumor cell death while sparing healthy tissues [340]. Additionally, nanoparticles can deliver radioprotective agents to normal tissues, reducing collateral damage. Preclinical studies have demonstrated that cerium oxide nanoparticles can scavenge reactive oxygen species, protecting healthy tissues during radiotherapy [341].

NBTXR3 hafnium oxide crystalline nanoparticles were evaluated in a pivotal multicenter phase II/III randomized trial (NCT02379845) comparing intratumoral NBTXR3 injection activated by preoperative radiotherapy versus radiotherapy alone in patients with locally advanced soft tissue sarcoma of the extremity and trunk wall. This clinical trial demonstrated a pathological complete response (pCR) rate of 16.1% in the NBTXR3 arm versus 7.9% in the radiotherapy-alone arm (OR 2.26; 95% CI: 0.98–5.02) [342]. NBTXR3 is further being evaluated in combination with anti-PD-1 therapy across multiple solid tumor types (NCT03589339) [253] and in locally advanced pancreatic cancer (NCT04484909).

Challenges include ensuring uniform nanoparticle distribution within tumors and optimizing timing with radiotherapy schedules. Future innovations may involve smart nanoparticles that release radioprotective agents in response to radiation-induced signals, further reducing side effects. These advancements could improve patient quality of life and expand the therapeutic window for radiotherapy.

### AI and nanotechnology

#### Role of AI in designing nanoparticles

The rational design of nanoparticles for cancer immunotherapy demands rapid, accurate prediction of physicochemical properties that govern in vivo performance. Supervised machine learning (ML) approaches—including random forests (RF), support vector machines (SVM), and deep neural networks (DNNs)—have been systematically applied to predict particle size, PDI, zeta potential, and encapsulation efficiency from formulation and process parameters; a two-stage ML pipeline combining a time-series model with a downstream regressor achieved a 7.5 × speedup in predicting nanoparticle solvent-accessible surface area (SASA)—a critical determinant of protein corona formation and cellular uptake—compared to full molecular dynamics (MD) simulation, with a mean absolute error of 1,956.93 Å^2^ across test systems [343]. A comprehensive survey of these methods demonstrated that ML models trained on historical formulation datasets can reliably extrapolate to novel lipid or polymer compositions, enabling in silico screening of hundreds of candidate formulations before any laboratory synthesis is performed and substantially reducing the number of empirical iterations required during development [344]. These advances collectively establish supervised ML as a practical first-pass filter in nanoparticle formulation pipelines, accelerating the transition from candidate generation to experimental validation.

MD simulation provides atomistic-resolution insight into nanoparticle-membrane interactions, lipid conformational dynamics, and self-assembly pathways that are inaccessible to empirical screening alone. Multiscale modeling frameworks—spanning atomistic MD, coarse-grained representations, and continuum methods—have been used to map self-assembly and membrane-translocation free-energy landscapes for polymeric and lipid nanocarriers, with MD-derived descriptors feeding ML predictors for emergent properties such as stability, drug release rate, and protein binding affinity [345]. A landmark demonstration of MD-ML integration used all-atom MD to derive dynamic conformational density images of ionizable lipid candidates during phase transitions; an ML classifier trained on these density images identified lipid P1, which adopted a three-tail cone conformation that promoted IgM protein corona formation and enabled selective spleen-targeted mRNA delivery with potent antibody and T cell responses in preclinical tumor models [346]. In the context of photoimmunotherapy, molecular simulation was used to guide the structural design of a self-adjuvanting nanoamplifier, informing nanoparticle-membrane interaction geometry and assembly conditions that were subsequently validated by photothermal and immunogenicity assays [347]. Together, these studies underscore that MD-ML integration is not merely a computational convenience but a mechanistically grounded strategy for rationally navigating the vast design space of therapeutic nanoparticles.

Generative artificial intelligence has emerged as a transformative paradigm for de novo discovery of nanoparticle components that would be challenging to identify through conventional screening. A generative pre-trained transformer (GPT) pipeline coupled to predictive ML scorers was used to design novel ionizable lipids for mRNA-based cancer immunotherapy; the top-ranked candidate, RNAi-PMA, produced a 10.6-fold increase in antigen-specific IgG titers and a fivefold increase in IFNγ-positive splenocyte counts relative to the benchmark lipid SM-102, while the overall discovery timeline was reduced by an estimated 80% compared to traditional empirical approaches [348]. Beyond lipid design, deep learning has been applied to the broader challenge of identifying co-assembling natural compounds for excipient-free nanoparticle formulation: a platform termed Gramord screened 1,800 naturally derived small molecules for co-assembly compatibility and identified the Ori-Cep pair (OCN), which accumulated at CT26 tumor sites, induced tumor regression, and promoted immune cell infiltration in murine models [349]. At the architectural level, generative adversarial networks (GANs) have been used to propose novel lipid structures that maintained or improved encapsulation efficiency, graph neural networks (GNNs) have predicted RNA-LNP binding affinity with high experimental concordance, and digital twin models applied to lipid nanoparticle lyophilization reduced formulation optimization timelines from years to months; minimum dataset sizes of approximately 15,000 lipid structures and model interpretability thresholds (SHAP > 0.65) are recommended for robust generalization in these workflows [350]. These results establish generative AI as a practical tool for accelerating the discovery of immunologically active nanocarriers.

Realizing the full potential of nano-immunotherapy requires aligning nanoparticle design with the molecular heterogeneity of individual tumors. Computational workflows that integrate multi-omics data—including genomics, transcriptomics, and proteomics—with nanoparticle property models enable co-optimization of neoantigen selection and nanocarrier formulation for personalized mRNA nanovaccines, linking sequence-level bioinformatics pipelines to LNP formulation optimization using molecular modeling tools [351]. For gold nanoparticles (AuNPs), computational screening and model-guided surface functionalization have been used to improve targeting specificity and immune modulation, providing a translatable framework for AuNP-based immunotherapy platforms [352]. AI-driven strategies that integrate multi-omics profiles with nanoparticle delivery parameters have been proposed to tailor nanocarrier composition to TME features such as immune cell infiltration, stromal density, and metabolic state, with the goal of overcoming tumor drug resistance [353]. The integration of ML with multi-omics readouts has further been proposed for Prussian blue nanoparticle (PBNP) photothermal immunotherapy, enabling individualized treatment planning based on patient-specific immune response monitoring [354]. Collectively, these multi-omics-integrated computational approaches represent a paradigm shift from population-level to patient-level nanoparticle design, directly addressing the heterogeneity that has historically limited clinical translation of nano-immunotherapy.

The convergence of supervised ML, generative AI, MD simulation, and multi-omics integration represents a comprehensive computational ecosystem for nano-immunotherapy design. Across these methodologies, several cross-cutting principles have emerged: model training datasets of at least ~ 15,000 lipid or nanoparticle structures are recommended for robust generalization, model interpretability metrics such as SHAP values exceeding 0.65 are proposed as practical thresholds for trustworthy predictions, and federated learning architectures enable cross-institutional training on multi-site nanomedicine datasets without sharing proprietary formulation data—collectively addressing the data fragmentation that has historically limited single-institution ML model performance [355]. Active learning and self-driving laboratory frameworks represent a further step toward autonomous nanoparticle optimization: an autonomous flow platform guided by active transfer-learning and multitask Bayesian optimization has been demonstrated for protein nanoparticle synthesis, achieving accelerated convergence to target size and PDI specifications with substantially reduced experimental iterations compared to conventional design-of-experiments approaches [356]. At the manufacturing interface, digital twin technology has been successfully implemented for GMP-compliant lipid particle production: a hybrid mechanistic-surrogate model integrated with PAT and IT/OT architecture was used to control adjuvant particle critical quality attributes in real time, with an engineering run demonstrating robust process performance under GMP conditions—providing a validated blueprint for digital twin deployment in nano-immunotherapy manufacturing scale-up [357]. As these computational frameworks mature and gain regulatory acceptance, their systematic integration into GMP-compliant manufacturing workflows will be essential for translating computationally optimized nano-immunotherapy candidates into clinical-stage products, ultimately closing the gap between in silico design and bedside application.

#### Predicting therapeutic outcome using AI

AI can predict therapeutic outcomes of nanoparticle-based immunotherapies by analyzing patient data, tumor characteristics, and treatment responses. This predictive capability is critical for identifying responders and optimizing treatment regimens [358].

ML models can integrate genomic, proteomic, and imaging data to predict how patients will respond to nanoparticle-based therapies [359]. For example, AI has been used to identify biomarkers of response to nanovaccines, such as neoantigen load and T-cell receptor diversity [360]. Predictive models can also optimize dosing schedules, as demonstrated in preclinical studies of nanoparticle-delivered cytokines, where AI predicted optimal release kinetics to maximize T-cell activation [361].

Artificial intelligence-guided approaches to optimize nanoparticle-based immunotherapy are an emerging research direction. Machine learning models trained on multimodal tumor data (genomics, transcriptomics, imaging) are being integrated into clinical trial designs to identify predictive biomarkers of response to checkpoint inhibitor-nanoparticle combinations [362].

Challenges include ensuring model generalizability across diverse patient populations and validating predictions in clinical settings [363]. Future directions may involve real-time AI monitoring during trials, using wearable devices or imaging to adjust nanoparticle dosing dynamically. Integrating AI with single-cell sequencing could further refine predictive models, enabling truly personalized immunotherapy [364].

### Combination with other modalities

#### Integration of nanotechnology with gene editing

The combination of nanotechnology with gene editing technologies, such as CRISPR/Cas9, offers transformative potential for immunotherapy by enabling precise modification of immune cells or tumor cells [365].

Nanoparticles can deliver CRISPR/Cas9 components (e.g., guide RNA, Cas9 protein) to T cells or tumor cells, enhancing immunotherapy efficacy. For example, lipid nanoparticles delivering CRISPR/Cas9 to knock out PD-1 in T cells have shown enhanced CAR-T cell activity in preclinical models of solid tumors [366]. Similarly, nanoparticles targeting tumor cells to disrupt immunosuppressive genes, such as TGF-β, have improved immune responses in melanoma models [367].

CRISPR-based gene editing is being applied clinically to enhance CAR-T cell therapy. A phase I multicenter study (NCT05722418, CaMMouflage Trial) is evaluating CB-011, a CRISPR-edited allogeneic anti-BCMA CAR-T cell therapy, in patients with relapsed or refractory multiple myeloma. Early results confirm feasibility and a manageable toxicity profile [260].

Challenges include ensuring precise gene editing without off-target effects and optimizing nanoparticle delivery to specific cell types [368]. Future developments may involve multiplexed gene editing, where nanoparticles deliver multiple CRISPR components to target several genes simultaneously, enhancing therapeutic efficacy [369]. Advances in nanoparticle targeting could also improve delivery specificity, reducing risks associated with systemic gene editing [370].

#### Nanoparticles for microbiome targeted immunotherapy

The gut microbiome plays a critical role in modulating immune responses and immunotherapy efficacy [371]. Nanoparticles can deliver microbiome-modulating agents, such as probiotics or microbial metabolites, to enhance immunotherapy outcomes [372].

Nanoparticles can encapsulate microbial metabolites, such as short-chain fatty acids, to promote immune activation in the TME. For instance, polymeric nanoparticles delivering butyrate have been shown to enhance anti-PD-1 efficacy in preclinical models of colorectal cancer by promoting T-cell infiltration [373]. Nanoparticles can also deliver siRNA to modulate gut microbiota composition, reducing immunosuppressive bacterial populations [374]. These approaches leverage the gut-tumor axis to enhance systemic immune responses [375].

The intersection of microbiome modulation and nanoparticle-based immunotherapy is an emerging translational frontier. Preclinical studies have demonstrated that nanoparticles delivering microbial metabolites can modulate gut immune homeostasis and enhance checkpoint inhibitor activity [376]. Multiple ongoing studies are evaluating fecal microbiota transplantation (FMT) combined with checkpoint inhibitors, providing a clinical framework for future nanoparticle-microbiome combination approaches [377].

Challenges include understanding the complex interactions between the microbiome and immune system and ensuring nanoparticle stability in the gastrointestinal environment [378]. Future directions may involve personalized microbiome modulation, where nanoparticles are tailored to individual microbial profiles [379]. Integrating microbiome sequencing with nanoparticle design could optimize therapeutic outcomes [380].

### Roadmap for clinical translation

#### Strategies to overcome current barriers

The clinical translation of nanotechnology-based immunotherapies faces several barriers, including scalability, regulatory challenges, and cost. A strategic roadmap is essential to address these hurdles and accelerate clinical adoption.

Scaling up nanoparticle production while maintaining quality is a major challenge. Continuous-flow synthesis and automated manufacturing platforms can enhance scalability, as demonstrated in the production of lipid nanoparticles for mRNA vaccines [381, 382]. Collaborative efforts between academia and industry are critical for developing cGMP-compliant facilities. For example, the successful scale-up of liposomal doxorubicin (Doxil) provides a model for nanomedicine production [383].

Regulatory agencies require comprehensive data on nanoparticle safety, biodistribution, and efficacy. Standardized guidelines for nanoparticle characterization, such as those issued by the FDA, can streamline approval processes [384]. Adaptive trial designs, which allow modifications based on interim data, can accelerate clinical development, as seen in trials of CAR-T nanoparticles [309, 385]. Early engagement with regulators through pre-submission meetings can clarify requirements and expedite approvals [386].

The high cost of nanomedicine production limits accessibility, particularly in low-resource settings. Developing cost-effective platforms, such as lipid nanoparticles, and leveraging economies of scale can reduce costs [387]. Public–private partnerships can also fund translational research, as seen in the development of mRNA vaccines [388].

#### Importance of interdisciplinary collaboration

Interdisciplinary collaboration among oncologists, material scientists, immunologists, and regulatory experts is essential for advancing nanotechnology-based immunotherapy.

Collaborative Research: Integrating expertise from multiple fields can accelerate innovation. For example, material scientists can optimize nanoparticle design, while immunologists can validate immune activation mechanisms. Collaborative platforms, such as the National Cancer Institute’s Nanotechnology Characterization Laboratory, have facilitated the development of clinically viable nanomedicines [389].

Translational Pipelines: Establishing pipelines that connect basic research, preclinical testing, and clinical trials is critical. Interdisciplinary teams can bridge these stages by combining expertise in nanoparticle synthesis, immune profiling, and clinical trial design. For instance, the development of NBTXR3 involved collaboration between material scientists and radiation oncologists, leading to successful phase III trials [390].

Global Networks: International collaborations can harmonize regulatory standards and share resources, accelerating global access to nanomedicines. Initiatives like the International Nanomedicine Consortium are fostering such networks, promoting the translation of nanoparticle-based immunotherapies [391].

The future of nanotechnology-based cancer immunotherapy is bright, with emerging technologies, AI integration, novel combinations, and strategic translational efforts paving the way for significant advancements. Nanoparticles for crossing the BBB, enhancing tumor visualization, and mitigating radiotherapy side effects hold promise for addressing unmet clinical needs. AI-driven nanoparticle design and outcome prediction are revolutionizing precision medicine, enabling personalized and optimized therapies. Combining nanotechnology with gene editing and microbiome modulation offers synergistic approaches to enhance immunotherapy efficacy. A strategic roadmap, supported by interdisciplinary collaboration, can overcome translational barriers, ensuring that these innovations reach patients. As nanotechnology continues to evolve, it is poised to redefine cancer immunotherapy, offering hope for improved outcomes in even the most challenging malignancies.

## Conclusion

Nanotechnology has emerged as a transformative force in cancer immunotherapy, addressing critical challenges such as low response rates, systemic toxicities, and TME barriers. This review has explored how nanotechnology enhances immune checkpoint inhibitors, cancer vaccines, CAR-T cell therapies, cytokine delivery, and TME modulation, while also examining clinical translation, regulatory hurdles, and future directions. By enabling targeted delivery, controlled release, and immune activation, nanoparticles may improve the therapeutic index of immunotherapies, as suggested by preclinical evidence.

Advances in nanoparticle-based immunotherapy include improved delivery of anti-PD-1/PD-L1 antibodies, enhanced antigen presentation in nanovaccines, in vivo CAR-T engineering, controlled cytokine release, TME reprogramming, and nanoparticle-assisted engineering of immune-cell therapies such as CIK cells. Notably, nanoparticle-based engineering of CIK cell immunotherapy represents a promising strategy to enhance localized immune-cell programming, sustain cytokine-mediated support, and improve antitumor function while addressing key limitations of conventional approaches. This emerging platform may provide a scalable and adaptable framework for the development of next-generation CIK-based cancer immunotherapies. Clinical trials have demonstrated the feasibility and efficacy of several nanoparticle-based immunotherapeutic approaches in cancers such as melanoma and lymphoma, with reduced toxicities compared with conventional therapies. However, regulatory challenges, including scalability and safety, require standardized guidelines and collaborative efforts to ensure clinical adoption.

In future perspectives, highlighting nanoparticles for crossing the BBB, enhancing tumor visualization, and mitigating radiotherapy side effects is discussed. Considering the rapid advancements in AI and ML, personalized nanoparticle design based on the identification of patient-specific immune signatures is anticipated to become a promising strategy for optimizing nano-immunotherapy approaches. The integration of AI in nanoparticle design and outcome prediction, along with combinations with gene editing and microbiome modulation, promises personalized and synergistic therapies.

## Data Availability

No datasets were generated or analysed during the current study.

## Abbreviations

TME: Tumor microenvironment
NPs: Nanoparticles
CIK: Cytokine-induced killer cell
PD-1: Programmed cell death protein 1
PD-L1: Programmed death-ligand 1
CTLA-4: Cytotoxic T-lymphocyte-associated antigen 4
CAR: Chimeric antigen receptor
MHC: Major histocompatibility complex
TCRs: T cell receptors
IL-2: Interleukin-2
irAEs: Immune-related adverse events
TAMs: Tumor-associated macrophages
MDSCs: Myeloid-derived suppressor cells
EPR: Enhanced permeability and retention
NSCLC: Non-small cell lung cancer
ICIs: Immune checkpoint inhibitors
PET: Positron emission tomography
ECM: Extracellular matrix
PDAC: Pancreatic ductal adenocarcinoma
IFP: Interstitial fluid pressure
RES: Reticuloendothelial system
AUC: Area-under-the-curve
LRP1: Lipoprotein receptor-related protein 1
TfR: Transferrin receptor
CT: Computed tomography
EV: Extracellular vesicle
APCs: Antigen-presenting cells
TLR: Toll-like receptor
LNPs: Lipid nanoparticles
IVT: In vitro Transcribed
PMAC: Membrane attack complex
CBI: Complement-based immunotherapy
PLGA: Poly (lactic-co-glycolic acid)
CoMiX: Complement-activating multimeric immunotherapeutic complexes
NK: Natural killer cell
DC: Dendritic Cell
ISCC: International Society of CIK Cells
dsDNA: Double-stranded DNA
cGAMP: Cyclic GMP-AMP
Treg: Regulatory T cell
CLDCu: Copper-chitosan nanoparticles
MHT: Magnetic hyperthermia
MNPs: Magnetic nanoparticles
AMF: Alternating magnetic field
ICD: Immunogenic cell death
PEG: Polyethylene glycol
HSPs: Heat shock proteins
DAMPs: Damage-associated molecular patterns
mRNA: Messenger RNA
PTT: Photothermal therapy
ADCs: Antibody-drug conjugates
DAR: Drug-antibody ratios
T-DXd: Trastuzumab deruxtecan
SBRT: Stereotactic body radiotherapy
HNSCC: Head and neck squamous cell carcinoma
ORR: Objective response rate
DCR: Disease control rate
GMP: Good Manufacturing Practice
PDI: Polydispersity index
CQAs: Critical quality attributes
IPCs: In-process controls
QbD: Quality-by-Design
CPPs: Critical process parameters
CFDs: Computational fluid dynamics
DoEs: Design-of-experiments
PAT: Process analytical technology
ISO: International Organization for Standardization
CQA: Critical quality attribute
ABC: Accelerated blood clearance
CARPA: Complement activation-related pseudoallergy
FDA: Food and Drug Administration
EMA: European Medicines Agency
AI: Artificial intelligence
BBB: Blood-brain barrier
CNS: Central nervous system
LIFU: Low-intensity focused ultrasound
BATs: Bispecific-armed T cells
ICG: Indocyanine green
pCR: Pathological complete response
ML: Machine learning
RF: Random forests
SVM: Support vector machines
DNNs: Deep neural networks
SASA: Solvent-accessible surface area
MD: Molecular dynamics
GPT: Generative pre-trained transformer
OCN: Ori-Cep pair
GANs: Generative adversarial networks
GNNs: Graph neural networks
PBNP: Prussian blue nanoparticle
FMT: Fecal microbiota transplantation
CTLs: Cytotoxic T lymphocytes
